# Two essential criteria for transcription factor footprint discovery using V-plot

**DOI:** 10.1038/s44320-026-00242-5

**Published:** 2026-08-25

**Authors:** Qifan Zhang, Chenhuan Xu

**Affiliations:** 1 Department of Computational Biology, China National Center for Bioinformation, Beijing, China; 2 Beijing Institute of Genomics, Chinese Academy of Sciences, Beijing, China; 3 University of Chinese Academy of Sciences, Beijing, China

**Keywords:** Chromatin, Transcription & Genomics, Computational Biology

## Abstract

A recent study reported sequence biases in transcription factor (TF) footprinting data, and expressed concern about the accuracy in TF footprint discovery using V-plot analysis. Here we define two graphical parameters of V-plot as important criteria to implement into future analysis. Guaranteed by the criteria, V-plot still serves as a powerful and straightforward tool for TF footprint discovery from various chromatin accessibility sequencing datasets.

## Abstract (web summary)

This correspondence clarifies transcription factor footprinting biases using V-plot analysis and offers parameters to mitigate bias for future analyses of chromatin accessibility data.

## Graphical abstract

**Figure:**
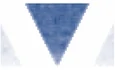

Using sequencing datasets for TF footprinting from our and others’ works (Carone et al, 2014; Henikoff et al, 2011; Hu et al, 2024; Michael et al, 2023; Schep et al, 2015), Durán et al, recently reported that the enzymes used in these methods can generate sequence biases at the fragmented DNA ends (Duran et al, 2026). Although such biases have been reported in previous studies (Allan et al, 2012; He et al, 2014; Wolpe et al, 2023), they found that the biases can generate V-shapes, when doing V-plot analysis, at genomic sites unoccupied by TFs, and expressed concern about the accuracy in identifying TF footprints by the combined presence of V-shapes and TF motif sequences.

The V-shape is roughly recognized by its geometric shape in routine V-plot analysis (Appendix Fig. S1). It is possible that the different V-shapes generated by enzyme digestion at bias sequences or at genuine TF occupancy sites may have distinct geometric characteristics. To investigate whether putative geometric differences can help distinguish V-shapes generated by TF occupancy or by bias sequences, we focused on sites with TF motifs closely juxtaposed with bias sequences. Using CTCF as an example TF and low dosage MNase digestion as the chromatin fragmentation method (loMNase-seq) (Hu et al, 2024), we performed V-plot analysis for three categories of genomic sites: (1 and 2) Highly or weakly occupied CTCF motifs with a juxtaposed MNase bias sequence, respectively and (3) MNase bias sequences without nearby CTCF motif (Fig. 1A). V-shapes appeared at all three categories of sites, but in distinct appearances. Notably, the V-shapes generated at occupied TF motifs are nested by an evidently wide V-channel vacant of signals, due to prohibited digestion within TF footprints under protection by TF occupancy (Fig. 1B). In contrast, the V-shape at MNase bias sequences exhibits a very slim V-channel (Fig. 1B). Additionally, the signals are apparently enriched within inside-V areas at occupied TF motifs, compared with evenly distributed signal intensity across inside-V and outside-V areas at MNase bias sequences (Fig. 1B), reflecting the enrichment of TF footprint-bearing fragments under MNase digestion. The enrichment within inside-V areas at occupied motifs is particularly evident from the V-shape generated using a CTCF ChIP dataset (Hu et al, 2024), but not at MNase bias sequences (Fig. 1C). DNA fragment end analysis also reveals a wide distance between the two flanking digestion “hotspots” at occupied motifs, indicative of a footprint region protected from digestion, which is absent at MNase bias sequences (Fig. 1D). We performed the same analysis using DNase-seq and ATAC-seq datasets, and the same graphical differences are observed between the V-shapes at occupied motifs and at DNase or Tn5 enzyme bias sequences respectively (Appendix Figs. S2 and S3).

**Figure 1:**
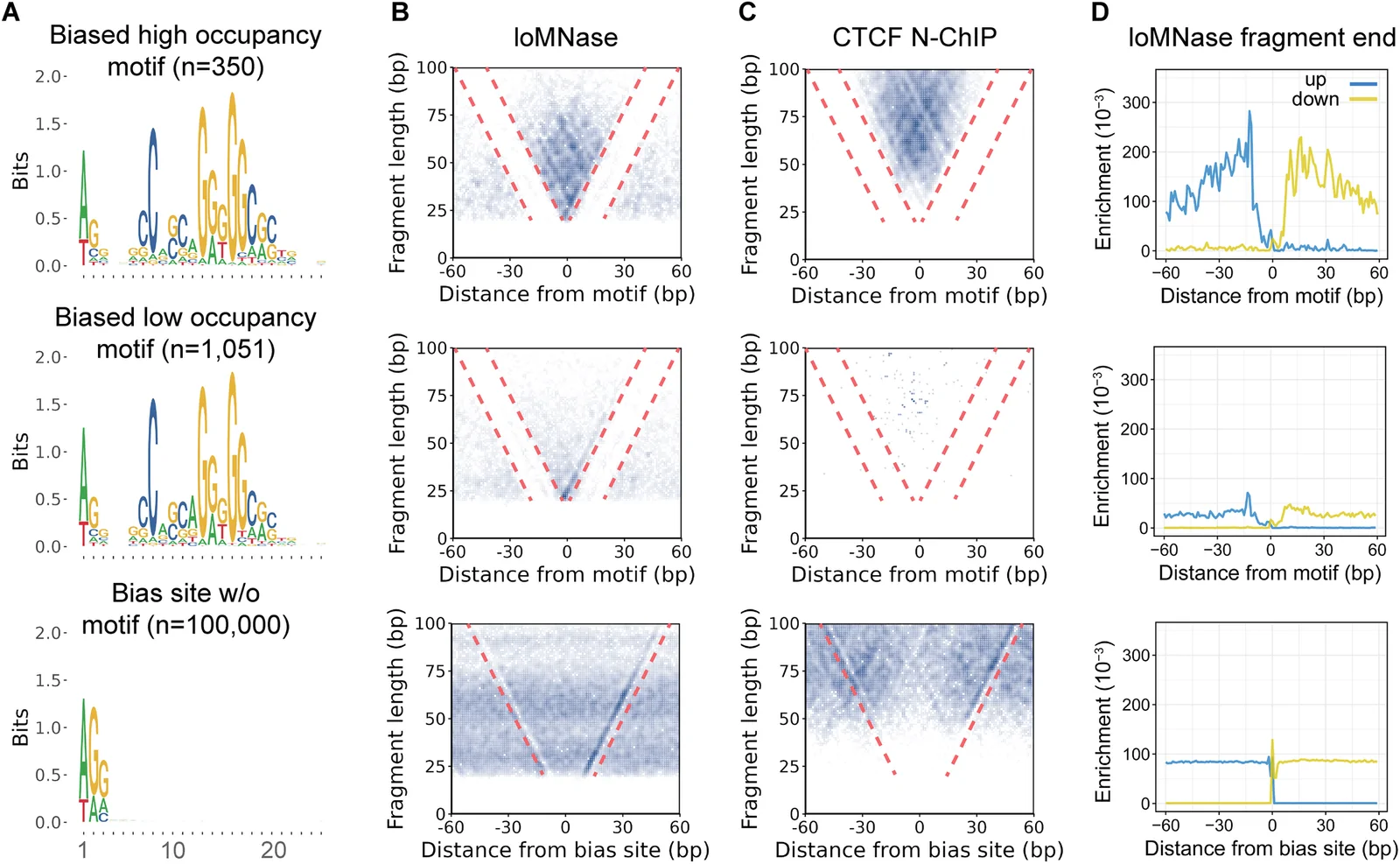
Occupied TF motifs and MNase bias sequences exhibit distinct V-plot features. (**A**) Sequence logos of highly or weakly occupied CTCF motifs preceded by a MNase bias sequence, and MNase bias sequences without a nearby CTCF motif. (**B**,** C**) V-plots of K562 loMNase-seq (**B**) or CTCF N-ChIP (**C**) fragments at three groups of sites defined in (**A**). Red dashed lines denote the V-channel. (**D**) The two ends of each loMNase-seq fragment are defined as upstream or downstream according to the CTCF motif or bias sequence directionality. The average profiles of upstream and downstream ends are centered around three groups of sites defined in (**A**).

In V-plot analysis, the length of each DNA fragment is plotted against the distance between the fragment center and a TF motif set of interest (Appendix Fig. S1). The inside-V and outside-V areas are filled by fragments with intact footprints or without any remnant footprint, respectively. Highly occupied motifs are reflected, in V-plot analysis, by high and low density of fragments within the inside-V or outside-V areas, respectively (Appendix Fig. S1). Additionally, in principle, the horizontal width of the V-channel vacant of signals should equal the minimal size of footprint, which is the exact region stringently protected by TF occupancy (Appendix Fig. S1). By following these rules, we performed an in silico digestion of MNase, and largely reproduced the wide V-channel and the inside-V enrichment of fragments at occupied CTCF motifs (Fig. 2A). Using MAZ as another TF example, we calculated the inside-V enrichment for V-shapes at each individual MAZ motif, and categorized them into five groups by the enrichment. The degree of enrichment highly correlates with MAZ occupancy level (Fig. 2B). Interestingly, the V-channel width seems to be constant, irrespective of the degree of inside-V enrichment (Fig. 2B), suggesting minimal footprint length as an intrinsic feature of TFs under certain digestion conditions.

**Figure 2:**
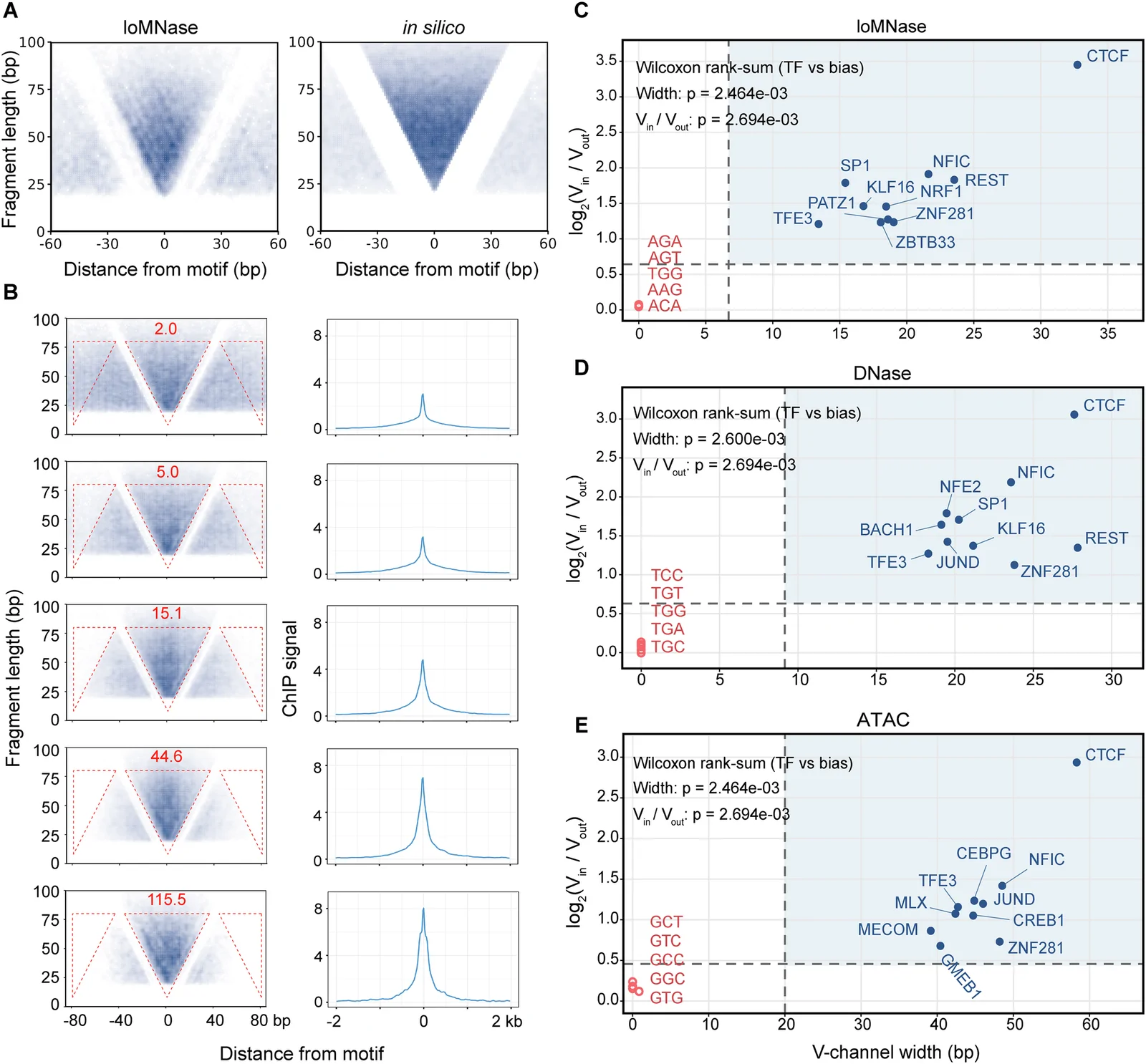
Two criteria for TF footprint discovery using V-plot. (**A**) V-plots of fragments from K562 loMNase-seq (left) or in silico MNase digestion (right) at CTCF motifs. (**B**) For each individual MAZ motif, the (V_in_ / V_out_) ratio of loMNase-seq fragments is calculated from the three triangle areas marked by red dashed lines. The area of the inside-V triangle equals the summed area of the two outside-V triangles. The MAZ motifs are divided into five groups by the ratio. Left: V-plots of loMNase-seq fragments at each group of motifs. Right: Average profiles of MAZ ChIP signals for each group of motifs. The red numbers show the mean ratio value for motifs within each group. (**C**–**E**) Scatter plots showing segregation of V-shapes at ten TF motifs from V-shapes at five bias sequences by (V_in_ / V_out_) ratio and V-channel width as two parameters, using loMNase-seq (**C**), DNase-seq (**D**), or ATAC-seq (**E**) data. Dashed lines denote segregation cutoffs for the two parameters. Red open circles indicate bias sequences, and blue filled circles indicate TF motifs.

Using the above principles, we next developed a pipeline to statistically examine the differences between V-shapes at occupied TF motifs and at bias sequences (Appendix Fig. S4). By the two graphical parameters, the V-channel width and inside-V enrichment, the V-shapes at different TF motifs can be statistically segregated from the V-shapes at bias sequences, for all three types of TF footprinting datasets (Fig. 2C–E; Appendix Figs. S5–7). Notably, different TFs have varying V-channel width (Fig. 2C–E), possibly due to their respective interfaces contacting DNA. For the same TF, different datasets exhibit relatively constant inside-V enrichment, but varying V-channel width (Fig. 2C–E), suggesting that footprint length is also subject to experimental conditions like enzyme kinetics. We also generated sets for shuffled or unoccupied CTCF motifs, and found that the inside-V enrichment alone can segregate the V-shapes at occupied CTCF motifs from the ones at shuffled or unoccupied motifs, indicating its usage in segregation with long putative bias sequences (Appendix Fig. S8).

In summary, we conclude that V-plot analysis of TF footprinting datasets, especially when in lack of robust ChIP data, should implement the V-channel width and inside-V enrichment as two essential criteria, to guarantee the validity of discovered TF footprints.

## Supplementary information

Appendix

Peer Review File

## Data Availability

The data generated in this study is available in the following database: loMNase-seq: [Genome Sequence Archive] [HRA017804] (https://ngdc.cncb.ac.cn/gsa-human/browse/HRA017804). The computer code produced in this study is available in the following database: GitHub (https://github.com/diovs/V-plot-criteria).
