# Supplementary material for: Two essential criteria for transcription factor footprint discovery using V-plot
Source: Mol Syst Biol. 2026 Aug 25;22(11):1668–71. doi: 10.1038/s44320-026-00242-5 (PMC13728155; doi:10.1038/s44320-026-00242-5)
Supplement: Supplementary file 1 [file 44320_2026_242_MOESM1_ESM.pdf]

Appendix

Two essential criteria for transcription factor footprint discovery using V-plot

Qifan Zhang[1,2,3] and Chenhuan Xu[1,2,3]*

*Correspondence: xuchh@big.ac.cn

[1] Department of Computational Biology, China National Center for Bioinformation, Beijing, China

[2] Beijing Institute of Genomics, Chinese Academy of Sciences, Beijing, China

[3] University of Chinese Academy of Sciences, Beijing, China

TABLE OF CONTENTS

Appendix Figure S1. A diagram illustrating the principle of TF footprint characterization using V-plot. 2

Appendix Figure S2. Occupied TF motifs and DNase bias sequences exhibit distinct V-plot features. 3

Appendix Figure S3. Occupied TF motifs and Tn5 bias sequences exhibit distinct V-plot features..... 4

Appendix Figure S4. A pipeline for locating V-shapes and determining (V_{in} / V_{out}) ratio and V-channel width. 5

Appendix Figure S5. V-plots of K562 loMNase-seq fragments at individual MNase bias sequences (A) or different TF motifs (B). 6

Appendix Figure S6. V-plots of K562 DNase-seq fragments at individual DNase bias sequences (A) or different TF motifs (B). 7

Appendix Figure S7. V-plots of K562 ATAC-seq fragments at individual Tn5 bias sequences (A) or different TF motifs (B). 8

Appendix Figure S8. Segregation of V-shapes at occupied CTCF motifs from scrambled or un-occupied motifs.......... 9

Methods 10

References 14

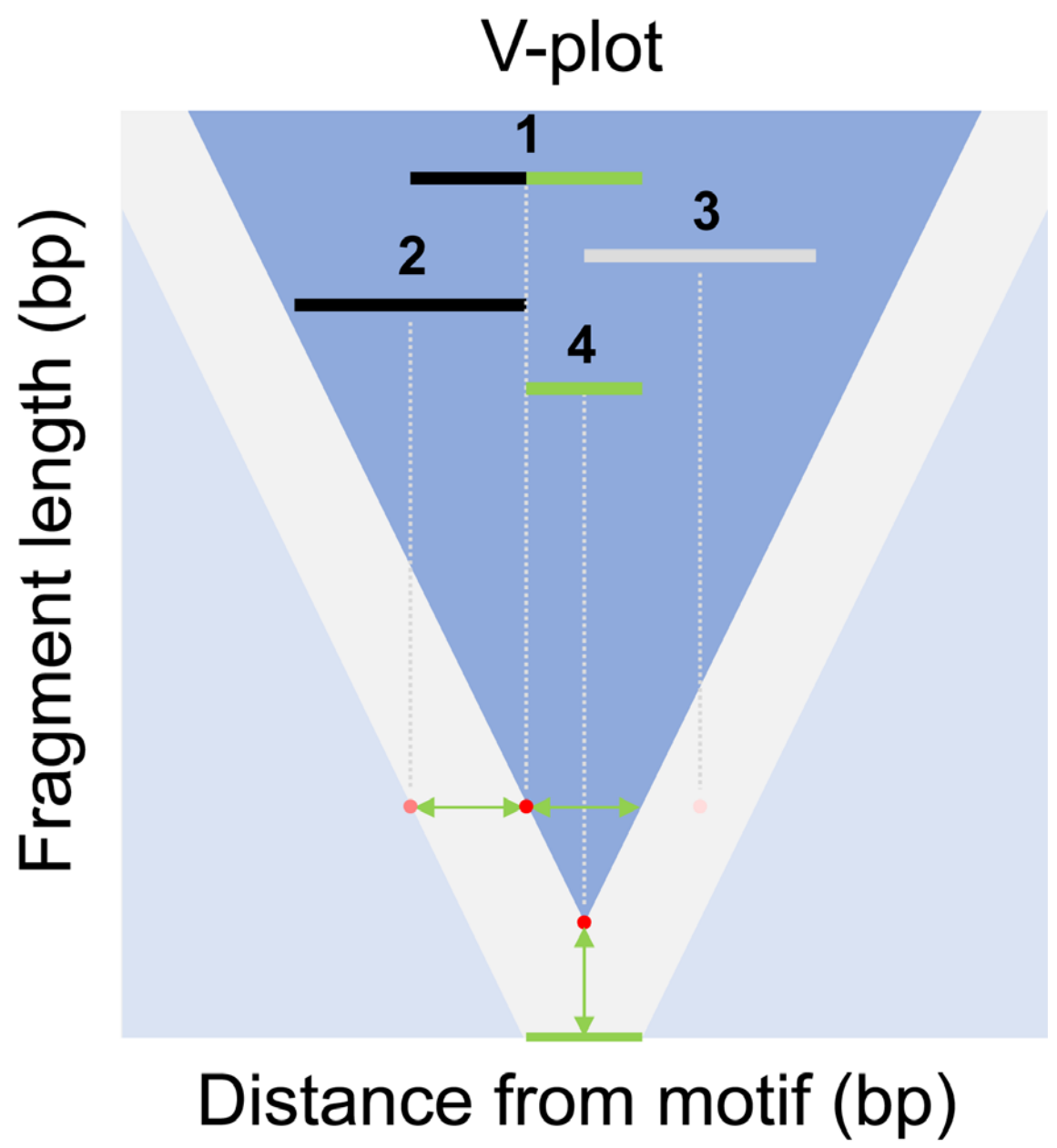

Appendix Figure S1. A diagram illustrating the principle of TF footprint characterization using V-plot. Four example fragments are shown. Fragments 1 and 4 include the footprint region. Fragment 2 excludes the footprint region. Fragment 3 is a hypothetical fragment containing a remnant of the footprint.

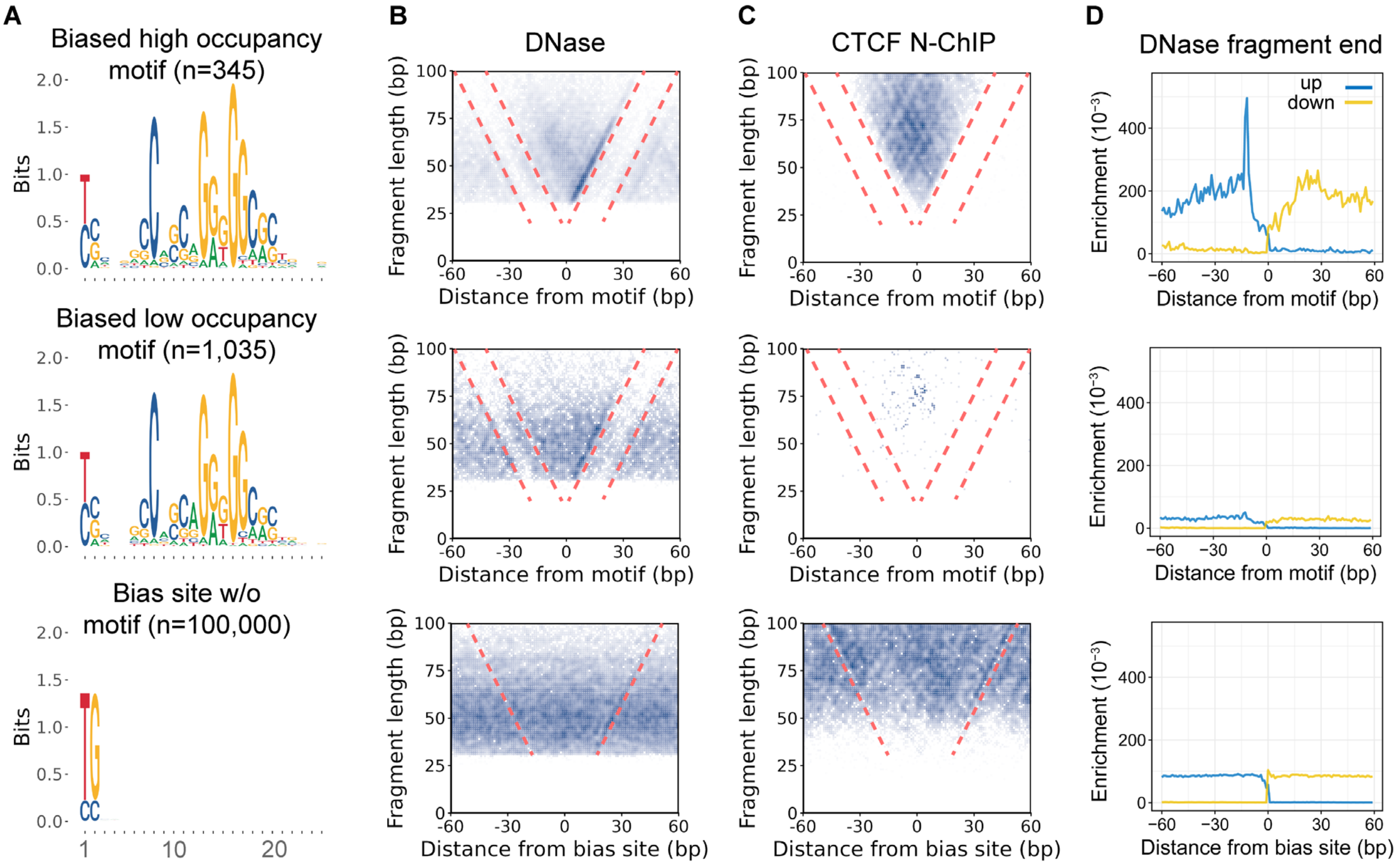

Appendix Figure S2. Occupied TF motifs and DNase bias sequences exhibit distinct V-plot features. **A** Sequence logos of highly or weakly occupied CTCF motifs preceded by a DNase bias sequence, and DNase bias sequences without nearby CTCF motif. **B and C** V-plots of K562 DNase-seq (**B**) or CTCF N-ChIP (**C**) fragments at three groups of sites defined in **A**. Red dashed lines denote the V-channel. **D** The two ends of each DNase-seq fragment are defined as upstream or downstream according to CTCF motif or bias sequence directionality. The average profiles of upstream and downstream ends are centered around three groups of sites defined in **A**.

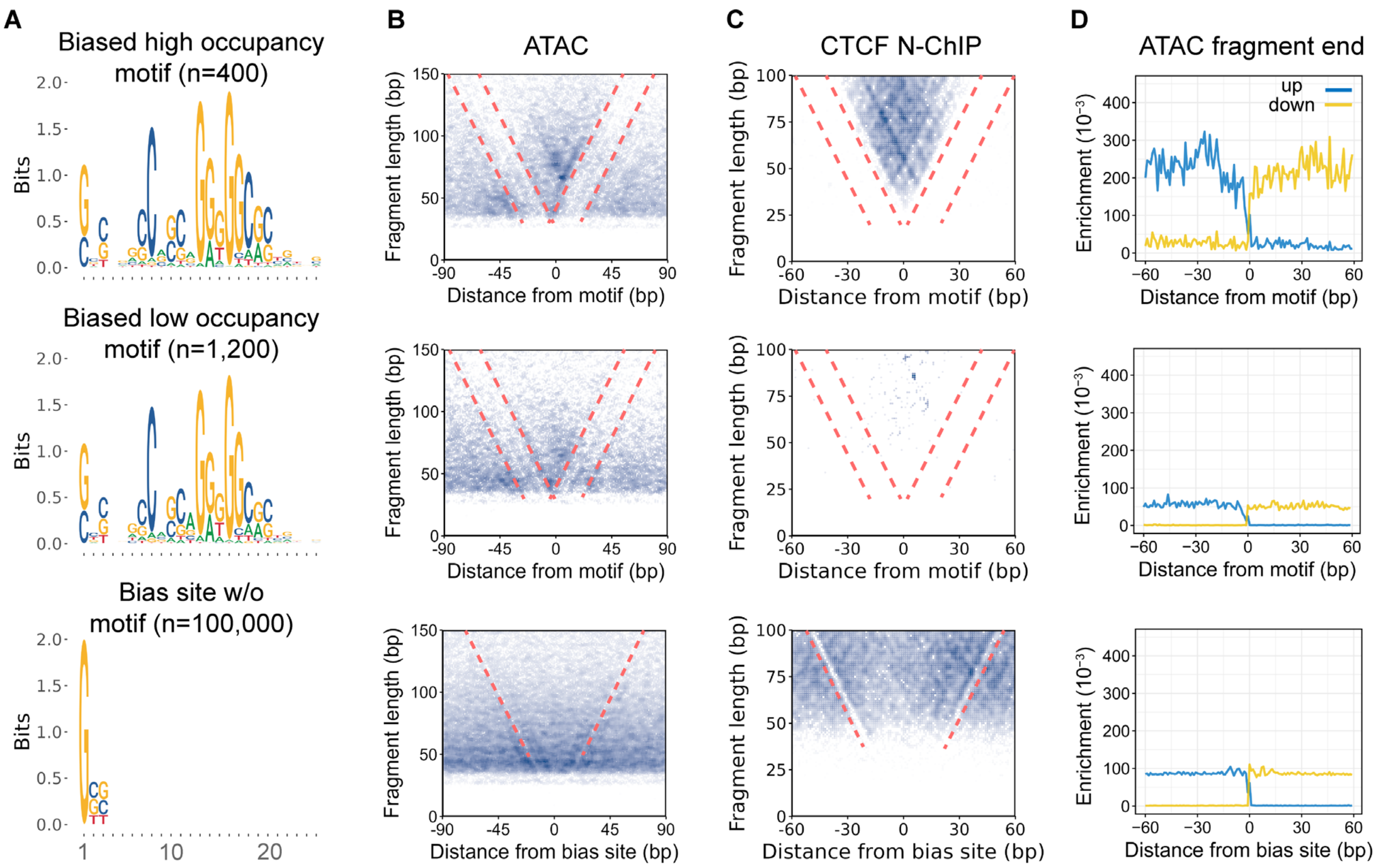

Appendix Figure S3. Occupied TF motifs and Tn5 bias sequences exhibit distinct V-plot features.
A Sequence logos of highly or weakly occupied CTCF motifs preceded by a Tn5 bias sequence, and Tn5 bias sequences without nearby CTCF motif. **B and C** V-plots of K562 ATAC-seq (**B**) or CTCF N-ChIP (**C**) fragments at three groups of sites defined in **A**. Red dashed lines denote the V-channel. **D** The two ends of each ATAC-seq fragment are defined as upstream or downstream according to CTCF motif or bias sequence directionality. The average profiles of upstream and downstream ends are centered around three groups of sites defined in **A**.

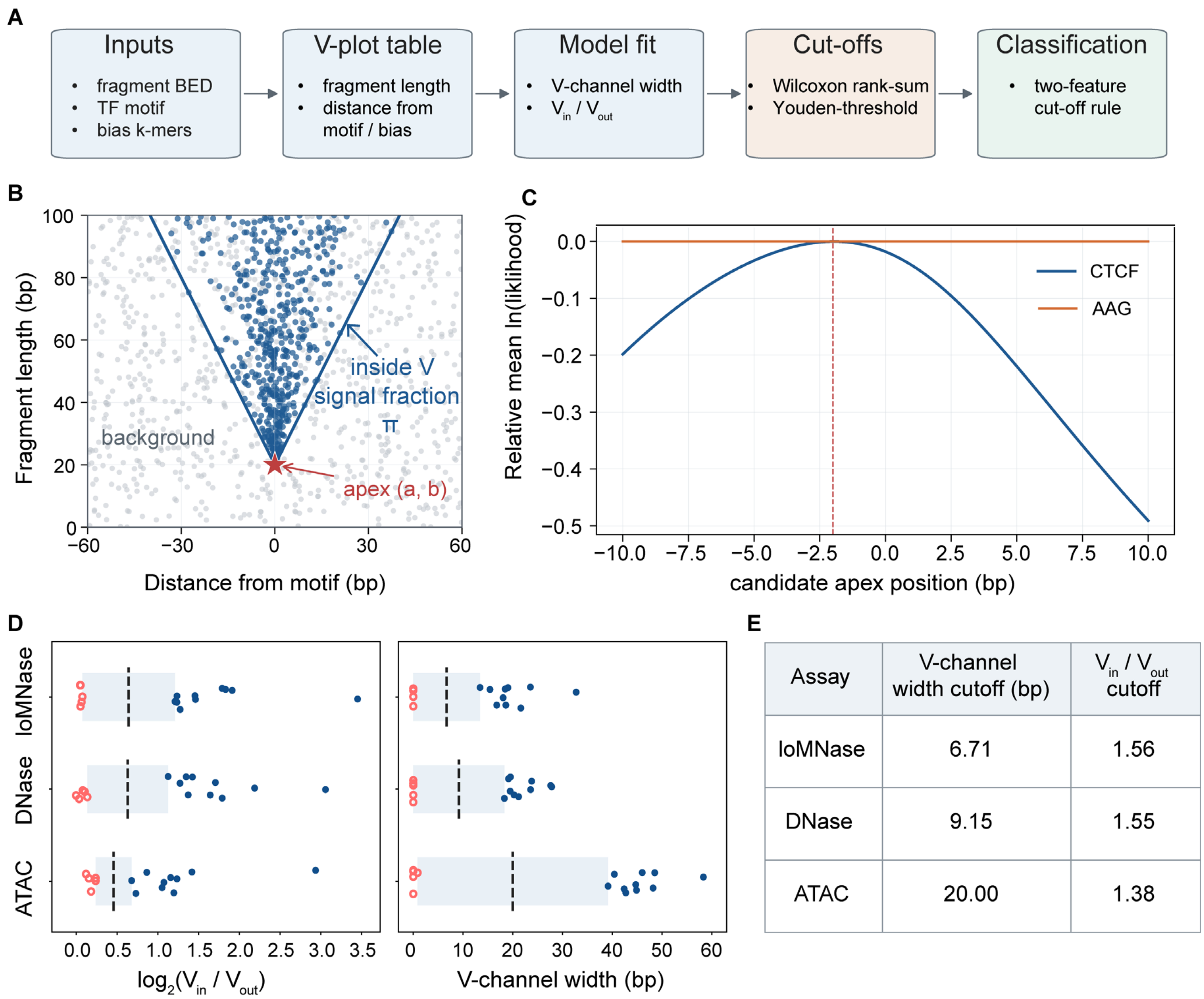

Assay	V-channel width cutoff (bp)	V_{in} / V_{out} cutoff
loMNase	6.71	1.56
DNase	9.15	1.55
ATAC	20.00	1.38

Appendix Figure S4. A pipeline for locating V-shapes and determining (V_{in} / V_{out}) ratio and V-channel width.

A Overview of the pipeline workflow. **B** Schematic of the V-plot model. The fitted apex (a, b) defines the V-channel width b, while fragments falling within the V-shaped region contribute to the inside-V signal fraction π; remaining fragments are modeled as background. **C** loMNase likelihood scan for CTCF and an AAG bias 3-mer. For each candidate apex position, the log-likelihood was calculated from the observed fragment distribution and shown relative to the maximum value for that curve. **D** Segregation of two parameters, (V_{in} / V_{out}) ratio (left) and V-channel width (right), for V-shapes at TF motifs and bias sequences, using loMNase-seq, DNase-seq, or ATAC-seq data. Blue filled circles indicate TF motifs, and red open circles indicate bias sequences. Dashed vertical lines mark the cutoffs, and shaded regions indicate the separation between bias and TF values. The table summarizes the segregation cutoffs for the two parameters.

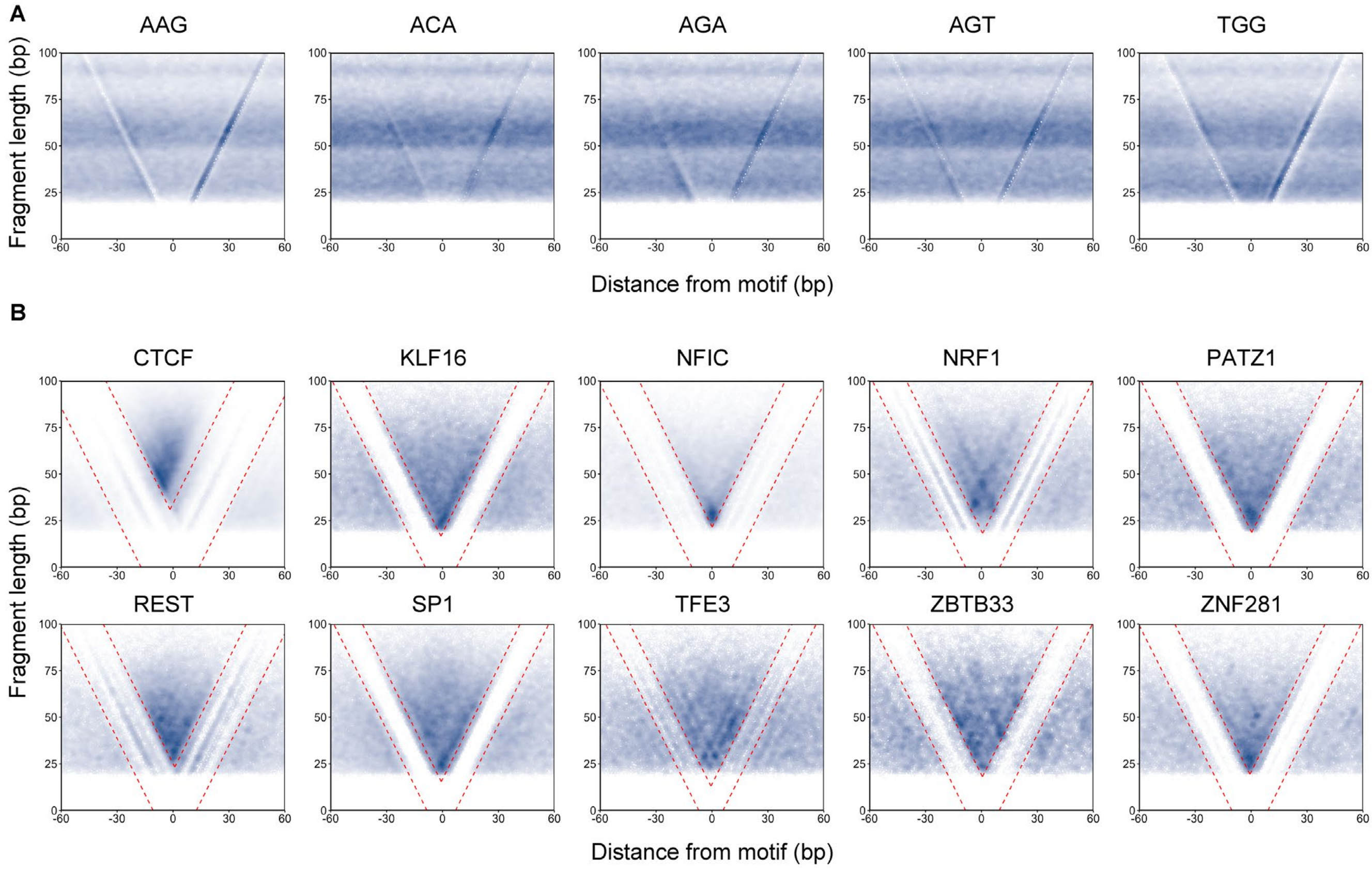

Appendix Figure S5. V-plots of K562 loMNase-seq fragments at individual MNase bias sequences (A) or different TF motifs (B).

Red dashed lines denote the V-channel.

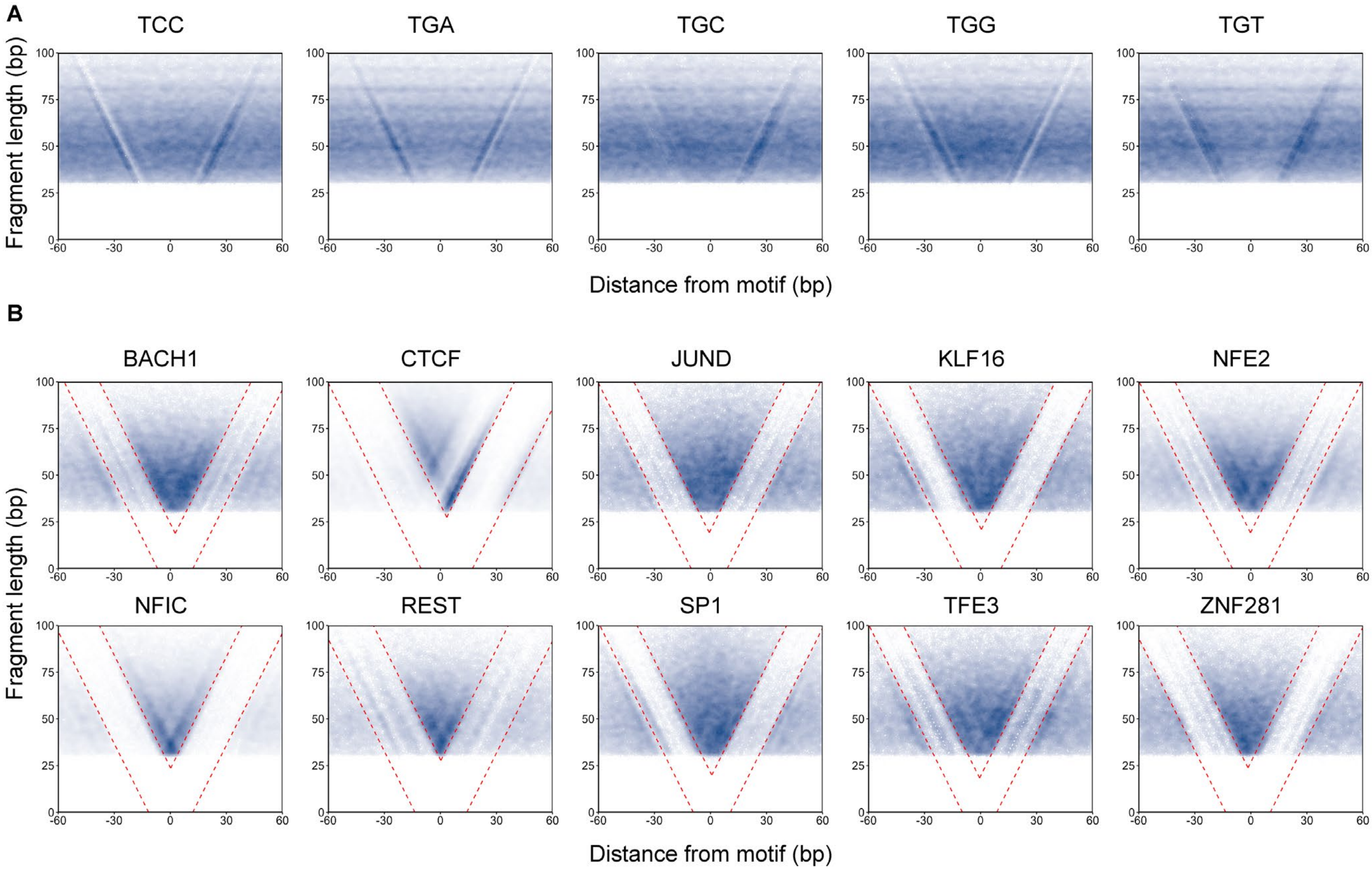

Appendix Figure S6. V-plots of K562 DNase-seq fragments at individual DNase bias sequences (A) or different TF motifs (B).

Red dashed lines denote the V-channel.

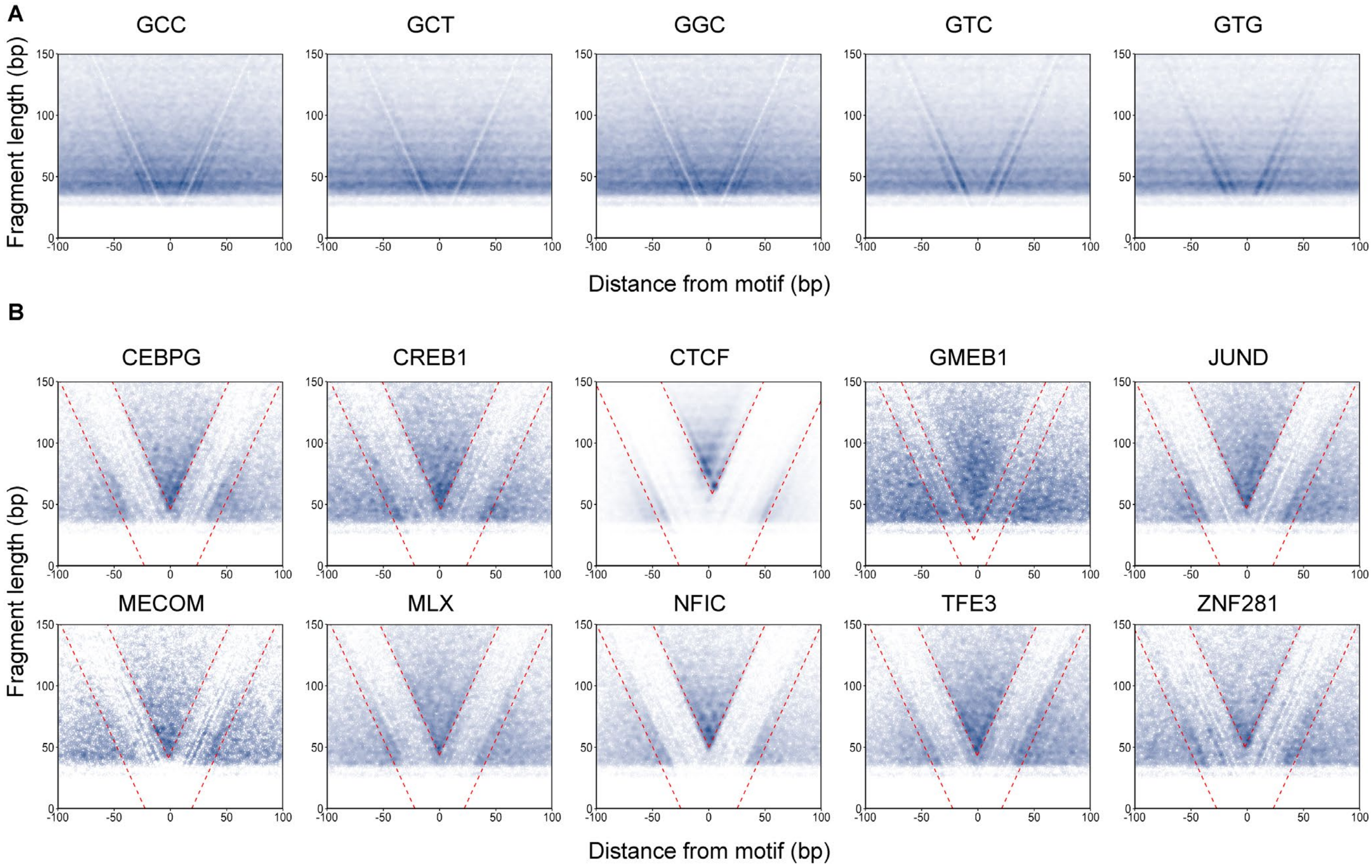

Appendix Figure S7. V-plots of K562 ATAC-seq fragments at individual Tn5 bias sequences (A) or different TF motifs (B).

Red dashed lines denote the V-channel.

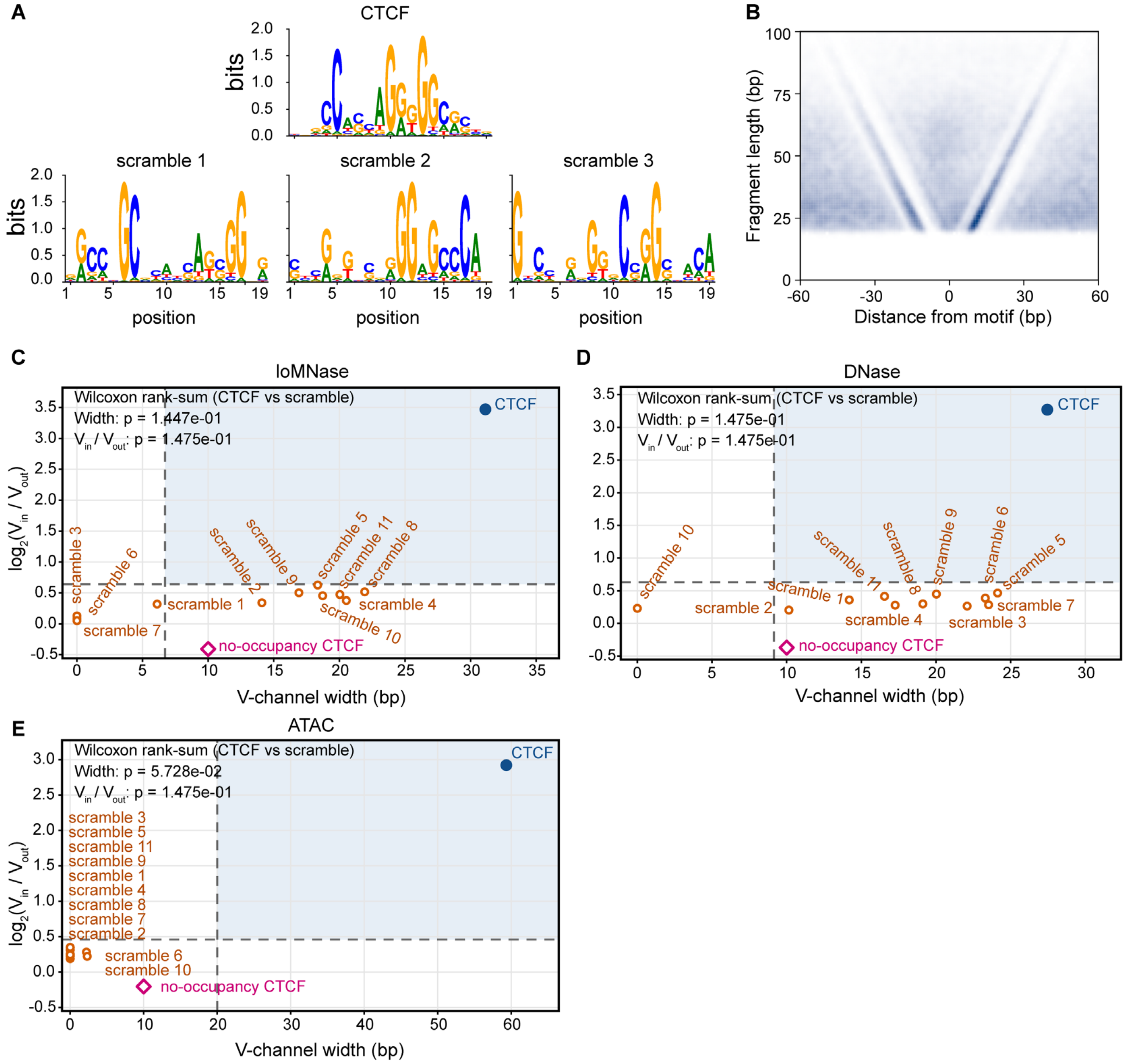

Appendix Figure S8. Segregation of V-shapes at occupied CTCF motifs from scrambled or un-occupied motifs.

A Sequence logos of the CTCF motif and three representative scrambled motifs. The nucleotides are scrambled without altering their weights at each position. **B** V-plots of K562 loMNase-seq fragments at un-occupied CTCF motif. **C-E** Scatter plots showing segregation of V-shapes at occupied CTCF motifs from V-shapes at un-occupied or 11 scrambled motifs by (V_{in} / V_{out}) ratio and V-channel width as two parameters, using loMNase-seq (**C**), DNase-seq (**D**), or ATAC-seq (**E**) data. Dashed lines indicate cutoffs for the two parameters. Orange open circles indicate scrambled motifs, blue filled circle indicates occupied CTCF motifs, and pink open diamond indicates un-occupied CTCF motifs.

Methods

Public data

Public data used in this study were obtained from ENCODE and public sequence repositories. K562 DNase-seq (ENCSR000EOT) and ATAC-seq (ENCSR483RKN and ENCSR868FGK) alignments were downloaded from ENCODE(Consortium *et al*, 2020). Transcription-factor occupancy was annotated with public ENCODE ChIP-seq data for the corresponding factors (Consortium *et al.*, 2020). The loMNase-seq and CTCF/MAZ native ChIP-seq (N-ChIP) data are available at the Genome Sequence Archive for Human (HRA005744 and HRA017804) (Hu *et al*, 2024).

loMNase-seq, ATAC-seq, and DNase-seq data analysis

Raw reads were subjected to trim_galore (version 0.6.10, [Babraham Bioinformatics - Trim Galore!]) to remove adapters and low-quality reads, then the trimmed reads were mapped to the hg38 genome using bowtie2 (version 2.5.1) (Langmead & Salzberg, 2012)with the options "*--very-sensitive --no-mixed --no-discordant --dovetail --score-min L,0,-0.4 -X 1000*". Reads aligned to the mitochondrial genome as well as reads with low mapping quality (MAPQ < 10) were discarded using SAMtools (version 1.6)(Li *et al*, 2009). Properly paired mates were converted to fragment intervals using the BEDTools toolkit (version 2.30.0) bamToBed tool (*-bedpe -mate1*) (Quinlan & Hall, 2010)and duplicate fragments sharing identical coordinates were removed. loMNase-seq and N-ChIP fragments were retained at ≤ 100 bp. For visualization, BEDTools genomeCoverageBed was used to create bedGraph files consisting of reads per million (RPM) values, which were converted to bigWig files using UCSC bedGraphToBigWig (version 2.8) (Kent *et al*, 2010). ATAC-seq and DNase-seq alignments were downloaded from ENCODE (Consortium *et al.*, 2020) and processed identically from the fragment-conversion step onward. Each fragment set was reduced to a six-column midpoint BED file (chromosome, midpoint, midpoint + 1, fragment index, fragment length, fragment index), which served as the input for all downstream V-plot analyses.

Transcription factor motif sources and annotation

Genome-wide motif coordinates were assembled from two complementary sources. Pre-called instances of HOMER (Heinz *et al*, 2010) known motifs for hg38 were taken directly from the HOMER bigBed track (homer.KnownMotifs.hg38.191020) without re-scanning. In addition, position frequency matrices from the JASPAR 2024 CORE vertebrate non-redundant collection (Rauluseviciute *et al*, 2024) were scanned against the hg38 genome using FIMO from the MEME suite (version 5.4.1) (Grant *et al*, 2011) under default settings (both strands; match $p < 1\times10^{-4}$; default background). To restrict analyses to genuinely bound sites, the motif instances of each factor were annotated with public ENCODE ChIP-seq signal averaged over a 100 bp window centered on the motif using UCSC

bigWigAverageOverBed (Kent *et al.*, 2010), and instances were ranked by this signal; the top 10% were retained as high-confidence bound sites.

Definition of biased high or low occupancy CTCF motifs

Each CTCF motif is bidirectionally expanded to 100 bp window, annotated using CTCF N-ChIP signal, and ranked by average signal across the window. High or low occupancy CTCF motifs are defined as the top 25% or bottom 75% ranked motifs. The two groups of motifs are respectively intersected with flanking bias sequences from loMNase-seq, DNase-seq, or ATAC-seq datasets respectively. The motifs with internal bias sequences are excluded from the two subsets.

Calculation of MAZ (V_{in} / V_{out}) ratio and occupancy level

One inside-V and two outside-V areas are defined as shown in Fig2B. The inside-V area equals the sum of two outside-V areas. The number of dots (or fragments) within inside-V or outside-V areas is counted, and the ratio of (V_{in} / V_{out}) is calculated for each MAZ motif. MAZ motifs are categorized into five groups if the ratio falls within 1-3, 3-9, 9-27, 27-81, or 81-max ranges. MAZ occupancy level shown in Fig2B is expressed in RPM (reads per million) values using MAZ N-ChIP data.

Enzyme bias sequence sites

For each assay (loMNase-seq, DNase-seq and ATAC-seq), the frequencies of the 3-nt sequences at fragment ends were tabulated and ranked, and the five most frequent end trinucleotides were taken as that enzyme's bias sequences. Genomic occurrences of each selected trinucleotide were located across the hg38 genome using seqkit (version 2.0.0) (Shen *et al*, 2016) locate and oriented by strand. Occurrences within ±12 bp of a CTCF motif were removed using BEDTools intersectBed (*-s -v*) to define bias sequences lacking a nearby TF footprint, and 100,000 sites were randomly sampled per trinucleotide (the same number for loMNase, DNase and ATAC) for the aggregate bias V-plots.

V-plot construction

For each anchor set (a TF motif set or a set of enzyme-bias sites), every fragment was assigned to its nearest anchor using BEDTools closestBed (*-d -t first*) on the midpoint BED, and the signed distance from the fragment midpoint to the anchor center (oriented by the anchor strand so that upstream and downstream are consistently signed) was recorded together with the fragment length; fragment–anchor pairs within a distance of 300 bp were retained. The V-plot is the resulting two-dimensional distribution of fragment length against the signed midpoint-to-anchor distance. V-plots were visualized as density heatmaps using the R package ggplot2 (version 3.4.4).

V-plot model pipeline for segregating TF footprints from enzyme bias

We developed a V-plot model pipeline to quantify whether a V-plot represents a TF-footprint-like pattern rather than enzyme cleavage bias. For each TF motif or matched bias sequence, the pipeline first generated a V-plot from fragment midpoint distances and fragment lengths, then fitted a simple V-shaped model to estimate two graphical features: V-channel width and inside-V enrichment (V_{in} / V_{out}). The fitted apex (a, b) defines the position of the V, with the y-coordinate b used as the V-channel width, corresponding to the length of the protected region. Inside-V enrichment was calculated as E = $\log_2$(fragment density inside the fitted V / fragment density outside the fitted V).

The pipeline also tested whether each V-plot contained a detectable V-like pattern by comparing the fitted V-plot model with a background-only model using a likelihood-ratio test, followed by Benjamini-Hochberg correction across motifs. When enabled, permutation calibration was performed by shuffling signed distances and refitting the model. This test was used to evaluate the presence of a V-like pattern, but not by itself to define a TF footprint, because enzyme-bias sequences can also generate V-like shapes.

To distinguish TF-footprint-like patterns from enzyme bias, the final pipeline step compared TF motifs with matched enzyme-bias controls using the two fitted features, V-channel width and V_{in} / V_{out}. Each feature was first tested by a two-sided Wilcoxon rank-sum test, and cut-offs were calibrated only when both features showed significant TF-versus-bias separation. For each feature, the cut-off was chosen as the Youden threshold, maximizing sensitivity + specificity - 1; when TF and bias distributions were fully separated, this was equivalent to the midpoint between the highest bias-control value and the lowest TF-motif value. A motif set was classified as TF-footprint-like only when both V-channel width and E exceeded their cutoffs.

***In silico* MNase digestion**

A global fragment length distribution is established using all fragments in loMNase-seq dataset. The *in silico* digestion is conducted under the assumption that only fragments either including complete or without any remnant CTCF motif sequence are generated. Each fragment length is randomly sampled from the global fragment length distribution described above.

Digestion boundary analysis

The 5′ and 3′ ends of each loMNase-seq fragment were extracted as single-base positions and oriented relative to the nearest CTCF motif or bias sequence. Strand-resolved upstream- and downstream-end coverage tracks were normalized to RPM values, and average profiles over a ±60 bp window centered on the anchor were computed at 1 bp resolution using the computeMatrix tool from the deepTools suite (Ramirez *et al*, 2016).

Sequence logos

Genomic sequences spanning each motif/bias element and its flanks were extracted using BEDTools getfasta (strand-aware) and rendered as information-content (bits) sequence logos using the R package ggseqlogo (Wagih, 2017).

Scrambled motifs

Length-matched negative control motifs were constructed from the CTCF motif. CTCF anchor positions were extended by ±9 bp (19 bp windows), strand-oriented, and tallied into a position-probability matrix, and the 19 position columns were randomly permuted (seeded), which preserves the per-position base composition and total information content while destroying their positional arrangement. The shuffled matrix was scanned across the hg38 genome with FIMO (version 5.4.1; $p < 1\times10^{-4}$; order-0 genome background), on which V-plots were generated for loMNase, DNase and ATAC.

Statistical analysis

Downstream quantification, modeling and statistical analysis were performed in Python (version 3.9.5; NumPy, pandas, SciPy and Matplotlib) and R (ggplot2 and ggseqlogo).

References

Consortium EP, Moore JE, Purcaro MJ, Pratt HE, Epstein CB, Shoresh N, Adrian J, Kawli T, Davis CA, Dobin A *et al* (2020) Expanded encyclopaedias of DNA elements in the human and mouse genomes. *Nature* 583: 699-710

Grant CE, Bailey TL, Noble WS (2011) FIMO: scanning for occurrences of a given motif. *Bioinformatics* 27: 1017-1018

Heinz S, Benner C, Spann N, Bertolino E, Lin YC, Laslo P, Cheng JX, Murre C, Singh H, Glass CK (2010) Simple combinations of lineage-determining transcription factors prime cis-regulatory elements required for macrophage and B cell identities. *Mol Cell* 38: 576-589

Hu S, Liu Y, Zhang Q, Bai J, Xu C (2024) A continuum of zinc finger transcription factor retention on native chromatin underlies dynamic genome organization. *Mol Syst Biol* 20: 799-824

Kent WJ, Zweig AS, Barber G, Hinrichs AS, Karolchik D (2010) BigWig and BigBed: enabling browsing of large distributed datasets. *Bioinformatics* 26: 2204-2207

Langmead B, Salzberg SL (2012) Fast gapped-read alignment with Bowtie 2. *Nat Methods* 9: 357-359

Li H, Handsaker B, Wysoker A, Fennell T, Ruan J, Homer N, Marth G, Abecasis G, Durbin R, Genome Project Data Processing S (2009) The Sequence Alignment/Map format and SAMtools. *Bioinformatics* 25: 2078-2079

Quinlan AR, Hall IM (2010) BEDTools: a flexible suite of utilities for comparing genomic features. *Bioinformatics* 26: 841-842

Ramirez F, Ryan DP, Gruning B, Bhardwaj V, Kilpert F, Richter AS, Heyne S, Dundar F, Manke T (2016) deepTools2: a next generation web server for deep-sequencing data analysis. *Nucleic Acids Res* 44: W160-165

Rauluseviciute I, Riudavets-Puig R, Blanc-Mathieu R, Castro-Mondragon JA, Ferenc K, Kumar V, Lemma RB, Lucas J, Cheneby J, Baranasic D *et al* (2024) JASPAR 2024: 20th anniversary of the open-access database of transcription factor binding profiles. *Nucleic Acids Res* 52: D174-D182

Shen W, Le S, Li Y, Hu F (2016) SeqKit: A Cross-Platform and Ultrafast Toolkit for FASTA/Q File Manipulation. *PLoS One* 11: e0163962

Wagih O (2017) ggseqlogo: a versatile R package for drawing sequence logos. *Bioinformatics* 33: 3645-3647
