# Supplementary material for: Two essential criteria for transcription factor footprint discovery using V-plot
Source: Mol Syst Biol. 2026 Aug 25;22(11):1668–71. doi: 10.1038/s44320-026-00242-5 (PMC13728155; doi:10.1038/s44320-026-00242-5)
Supplement: Supplementary file 2 [file 44320_2026_242_MOESM2_ESM.pdf]

Two essential criteria for transcription factor footprint discovery using V-plot

Qifan Zhang and Chenhuan Xu

Corresponding author(s): Chenhuan Xu (xuchh@big.ac.cn)

Review Timeline:

Submission Date:	20th May 26
Editorial Decision:	11th Jun 26
Revision Received:	10th Jul 26
Editorial Decision:	5th Aug 26
Revision Received:	6th Aug 26
Accepted:	13th Aug 26

Editor: Poonam Bheda

Transaction Report:

(Note: With the exception of the correction of typographical or spelling errors that could be a source of ambiguity, letters and reports are not edited. Depending on transfer agreements, referee reports obtained elsewhere may or may not be included in this compilation. Referee reports are anonymous unless the Referee chooses to sign their reports.)

11th Jun 2026

Manuscript Number: MSB-2026-13790
Title: Two essential criteria for transcription factor footprint discovery using V-plot
Author: Qifan Zhang
Chenhuan Xu

Dear Dr Xu,

Thank you again for submitting your work to Molecular Systems Biology. We have now heard back from the three reviewers who agreed to evaluate your Correspondence. As you will see below, the reviewers appreciate that you have qualitatively shown that there are indeed differences in V-plots from bona fide transcription factor binding sites compared with naked DNA. However, they provide some constructive feedback and suggestions, which we would ask you to address in a revision. One of the more fundamental points raised refers to the suggestion to provide practical information and guidance on how to implement your findings in V-plot analysis going forward, e.g. with statistical testing or cut-offs. All other issues raised should also be addressed. Please let me know in case you would like to discuss in further detail any of the any of the reviewer comments or your proposed revisions, I would be happy to schedule a call.

Yours sincerely,

Poonam Bheda, PhD
Senior Scientific Editor
Molecular Systems Biology

Reviewer #1:

The authors present a note on a recent paper by Durán et al. This recent paper argues against the use of V-plots for identifying protein-DNA interactions, as the same V-shapes could be reproduced from deproteinized DNA, due to the sequence bias of the digestion enzymes. Here, the authors follow up on this observation and investigate whether certain characteristics of the V-plot can still distinguish genuine protein footprints from sequence biases. They analyze different transcription factor motifs and accessibility assays, as well as an in silico experiment and find the V-channel width and inside-V enrichment to be the distinguishable characteristics.

Major:
The practical consequences of the observations are hard to estimate. While the authors show convincingly that there are differences in the V-plot between bias sequences and protein binding motifs, how this information can be used in practice remains open. It would be beneficial if there would be advice on cut-offs or a statistic on how to distinguish bias and genuine binding.
The TF motifs analyzed in Figure S3 and S4 are all CG-heavy. Durán et al. raised the point of differences in the V-plots due to varying backgrounds. Are the differences in V-plots presented here also valid for motifs with more A and T?

Minor:
Generally, more details on the methods would be beneficial: How were highly and weakly occupied CTCF sites defined in Figure 1? How was the in silico MNase digestion done? How were the MAZ occupancy levels defined?
Could there be an explanation added why motif sites with a juxtaposed bias sequence were analyzed and not all motif sites?
I couldn't find the datasets on GSA, could the accession codes be double checked?
I think the word "concerned" is not correctly used, e.g. 'further concerned about' should be 'expressed concern about' or 'were concerned about'.

Code:
The GitHub hyperlink link is not working, but findable via the displayed text.
While the GitHub folder structure makes it possible to navigate through the files, there is no README with an overall description.

Reviewer #2:

Summary

Zhang & Xu provide a compelling reply to Duran et al. (Genome Biol. 2026), who have recently provided seemingly straightforward but disputable evidence claiming that V-plots are not a reliable methodology to investigate protein-DNA interactions. Zhang & Xu clearly illustrate, both qualitatively and quantitatively, differences between "real" V-plot signals (obtained when focusing on TF binding motifs) and artifact V-plots (obtained when focusing on enzyme's bias motifs).

This manuscript supports the relevance of V-plots in epigenomic analysis workflows. This is of broader significance: conclusions from Duran et al. were raised questions about the validity of previously published results based on V-plot analyses. Zhang & Xu indisputably refute these conclusions to show that biologically relevant V-plots are valuable and can be quantified. Researchers who have used this approach in the past, or were intending to use it for future analyses, are thus reassured that V-plot-based interpretations remain methodologically sound and biologically meaningful, and can proceed with confidence in building upon or applying this framework in their epigenomic studies.

Major points

My main suggestion would relate to the size of the sequence the authors use to support their claim. They use 3bp-long sequence bias motifs for MNase, DNase or Tn5, and in fact recover the same type of non-significant V-plot signal (thin white lines, ratio of Vin/Vout of ~ 1). I would recommend generating V-plots centered on randomly chosen sequences with a length comparable to that of the TF motifs they are studying (7-16bp, according to Fig. 2D). Even though this would not directly address the question of the V-plot artifact emerging from sequence bias from enzymes, this would clarify the comparison between a well-resolved V-plot and a non-significant V-plot over motifs of the same size.

Although this goes beyond the review of this individual manuscript, I suspect that the MNase dataset generated in S. pombe by Duran et al., shown in their Fig. 1C, is in fact of insufficient quality to reliably identify V-plots. Duran et al. have also used a non-standard computational approach to annotate TF binding sites in the S. pombe genome. I believe it could be worth investigating the quality of their data, should the authors want to more directly and openly dispute conclusions from Duran et al.

In the same vein, it would be important to specify which tools were used in this study for motif scanning (e.g., FIMO, MEME, others?), and exact scanning parameters, as these choices can significantly influence the resulting V-plot signals.

Minor points

- A Methods section is missing. I understand that all analysis notebooks are publicly available, but I believe at least some basic information should be provided in a Methods section, at least to cite the libraries being used (e.g. bedtools, R, ggplot, etc) (and ideally their version).
- Why is the schematic explanation of V-plots in Fig. 2A? I would suggest having it in main Fig. 1 or in first Fig. S1, to clearly introduce to the reader what the plots represent.

Reviewer #3:

This commentary provides important insight and guidance for transcription factor footprint analysis using V-plots. The recent paper by Duran et al. suggested that a V-shaped pattern can be observed even in the absence of a true transcription factor footprint, due to sequence bias when motif-centered analysis is performed. The authors aim to provide criteria to distinguish artificial V-shaped patterns caused by sequence or enzyme bias from true transcription factor footprints. Using CTCF and other transcription factor motifs as examples, the authors demonstrate that a wider V-channel and inside-V enrichment are useful indicators for detecting true footprints. Although these criteria are potentially valuable, the results presented here remain largely qualitative. The manuscript does not provide statistical methods or quantitative measurements to support the conclusions or to standardize the proposed approach. Additional quantitative analyses would strengthen the claim that these features can reliably distinguish true transcription factor footprints from bias-driven artifacts. Other major concerns are summarized below.

Line 79: The authors state: "In summary, we conclude that V-plot analysis using chromatin accessibility datasets, especially when in lack of robust ChIP data, should implement the V-channel width and inside-V enrichment as two essential criteria, to guarantee the validity of discovered TF footprints."

This strong claim requires additional supporting evidence and quantitative measurements with a statistical test. Although Figure 2D shows a clear separation between TF target sites and 3-mer sites, the analysis does not report variability across biological replicates or across different data sources. Because ATAC-seq is the most widely used method and appears to produce the least discrete V-plot pattern, ATAC-seq data should also be included in Figure 2D to assess whether the observed separation is robust across assays.

Low-MNase-seq:

Low-MNase-seq is quite different from ATAC-seq and DNase-seq, and less popular compared to other methods. I am also not sure whether low MNase-seq should be categorized as a chromatin accessibility assay, although it has been used to capture

MNase-sensitive nucleosomes and transcription factor footprints. The data from standard MNase-seq would also be informative.

As shown in Supplementary Figure S2, the ATAC-seq data show weaker and more ambiguous footprints or V-shaped patterns. By comparing these three panels, it still appears challenging to draw a definitive conclusion based solely on the suggested criteria of V-channel width and inside-V enrichment.

Therefore, I believe the concerns raised by Duran et al. still provide an important caution for the field. V-shaped and enrichment patterns may differ depending on the combination of genomic data type, enzyme bias, and motif sequence composition. Sequence bias induced by MNase is also known to vary depending on MNase concentration.

Confirmation with other TFs and loci
It would also be useful for the authors to further clarify the choice of bias sites without motifs. The number of these negative control sites is also much larger than the number of CTCF binding sites, which may affect the comparison. For further validation, it would be useful to examine V-plots at genomic loci that contain CTCF motifs but are not bound by CTCF. Because MNase is known to prefer AT-rich sequences, it would also be important to assess V-plot bias at AT-rich sequences and at motifs for AT-rich sequence-binding transcription factors, as most of the transcription factors tested here appear to target GC-rich sequences.

We appreciate the dedication and helpful suggestions from all reviewers. Below we address your comments point-by-point in red text. All figure changes are summarized below:

New figures:

Fig. S4

Fig. S7

Fig. S8

Updated figures:

Fig. 2

Figure order changes:

Old Fig. 2A, now Fig. S1

Old Fig. S1, now Fig. S2

Old Fig. S2, now Fig. S3

Old Fig. S3, now Fig. S5

Old Fig. S4, now Fig. S6

Reviewer #1:

The authors present a note on a recent paper by Durán et al. This recent paper argues against the use of V-plots for identifying protein-DNA interactions, as the same V-shapes could be reproduced from deproteinized DNA, due to the sequence bias of the digestion enzymes. Here, the authors follow up on this observation and investigate whether certain characteristics of the V-plot can still distinguish genuine protein footprints from sequence biases. They analyze different transcription factor motifs and accessibility assays, as well as an in silico experiment and find the V-channel width and inside-V enrichment to be the distinguishable characteristics.

We thank you for your time and the summary of our work.

Major:

The practical consequences of the observations are hard to estimate. While the authors show convincingly that there are differences in the V-plot between bias sequences and protein binding motifs, how this information can be used in practice remains open. It would be beneficial if there would be advice on cut-offs or a statistic on how to distinguish bias and

genuine binding.

In the revised manuscript, we provide an executable pipeline (deposited at https://github.com/diovs/V-plot-criteria/tree/main) to help putative users examine the V-shapes generated from their datasets using statistical methods. In brief, users are prompted to specify the data type from one of IoMNase-seq, DNase-seq, or ATAC-seq, and to input the coordinates of TF motifs of interest and a *.bed* file including the coordinates for digested fragments from the above assays. The pipeline first examines the presence of V-shapes at input motif coordinates and if so, locates the V-shapes at single-base resolution, calculates V-channel width and (V_{in} / V_{out}) ratio using a likelihood-ratio test. Next, using bias sequences from IoMNase-seq, DNase-seq, or ATAC-seq data or any customized sequences, the pipeline examines if the two graphical parameters, the V-channel width and (V_{in} / V_{out}) ratio, are significantly different between the TF motifs and the bias sequences, using a Wilcoxon rank-sum test. Below we provide some TF examples for the three data types respectively, to show that V-shapes at TF motifs can be statistically segregated from the V-shapes at bias sequences by the two aforementioned graphical parameters (also as updated Fig2C-E).

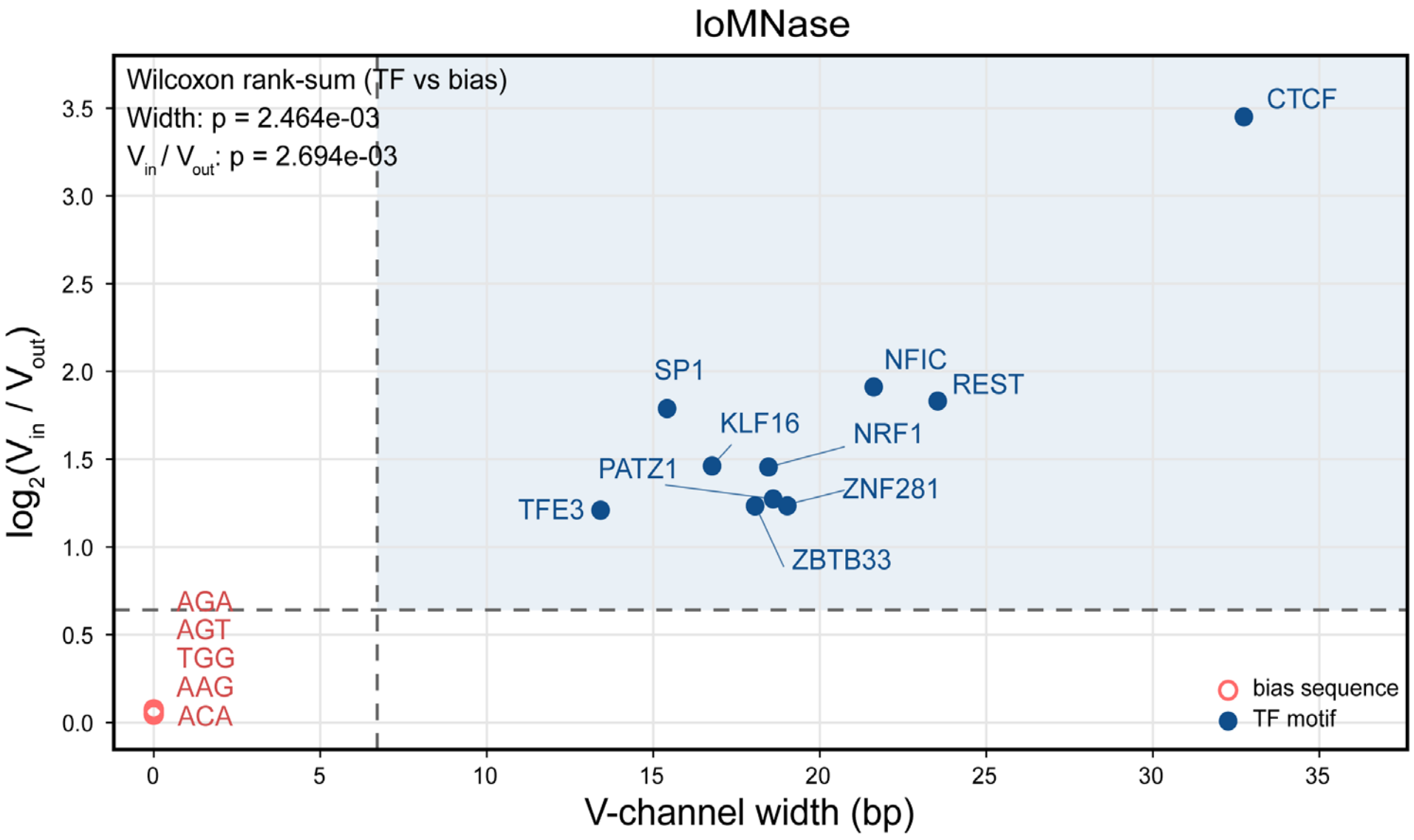

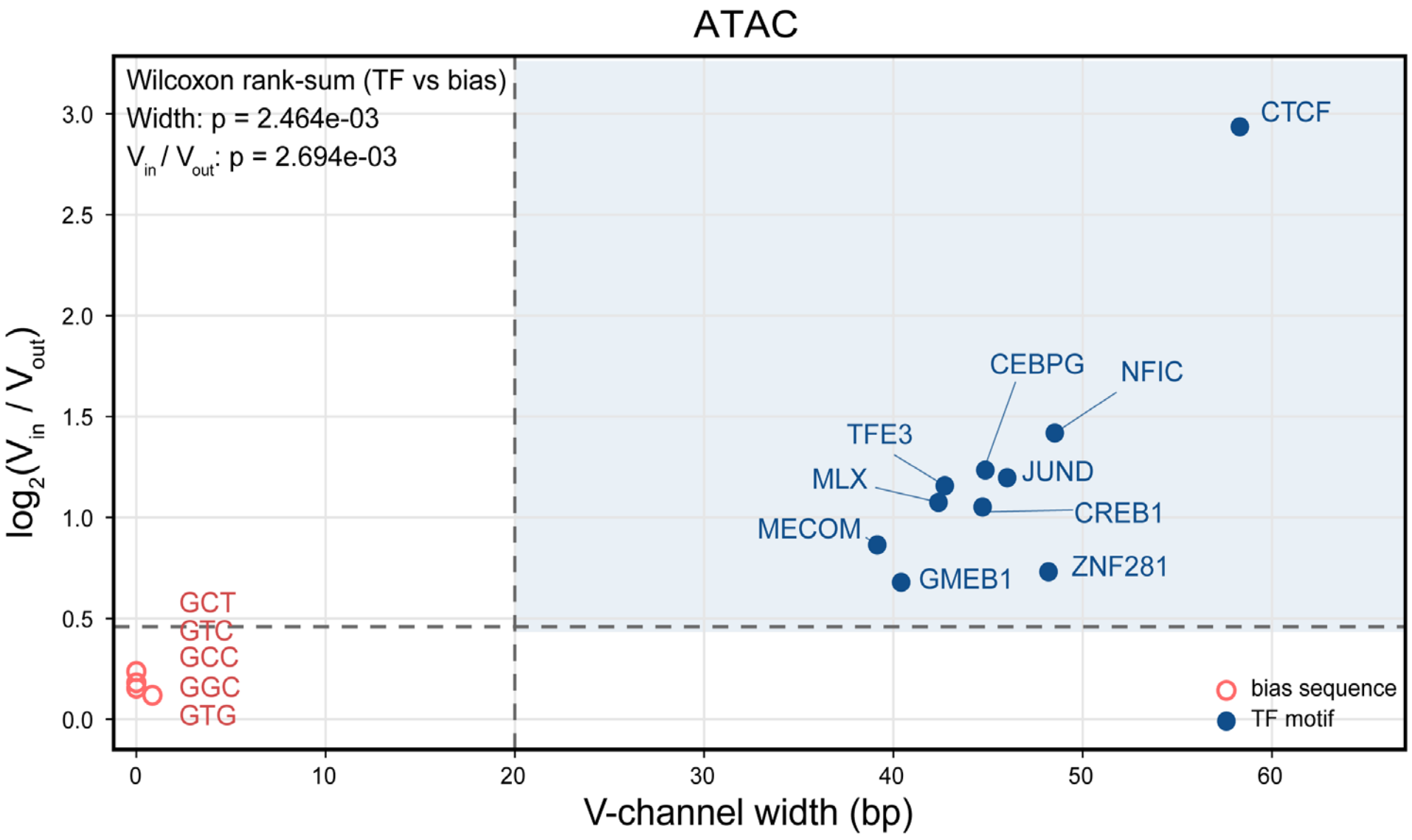

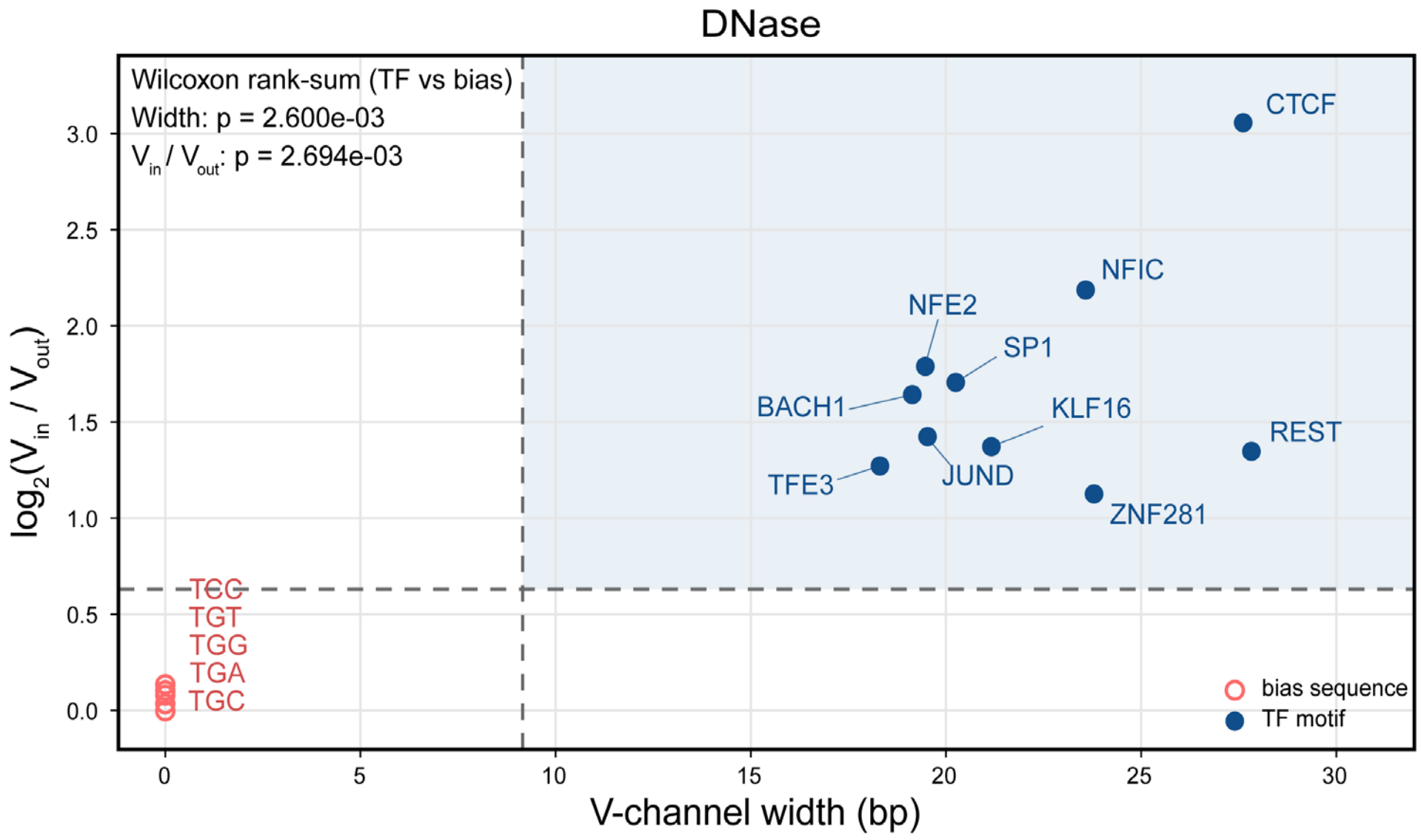

The pipeline determines the segregation cutoffs for the two graphical parameters using a Youden-threshold procedure. Below we show the cutoffs from the above three examples.

Assay	V-channel width cutoff (bp)	Vin / Vout cutoff
**loMNase**	6.7	1.56
**DNase**	9.2	1.55
**ATAC**	20.0	1.38

Detailed description of the pipeline is provided in the README file deposited at https://github.com/diovs/V-plot-criteria/tree/main.

The TF motifs analyzed in Figure S3 and S4 are all CG-heavy. Durán et al. raised the point of differences in the V-plots due to varying backgrounds. Are the differences in V-plots presented here also valid for motifs with more A and T?

In updated Fig2C-E, we include TFs with relatively balanced nucleotide composition in their motif sequences. The V-shapes at these motifs can be well segregated from the ones at bias sequences. Below we show a few TF examples for each of the three data types. It should be noted that GC content relates to TF-DNA interactions. It is known that zinc finger TFs (eg., CTCF, MAZ) favor GC-rich motif sequences (PMID: 26160164), and have relatively long residence time on chromatin. Therefore, some certain TFs may prefer GC-rich motifs and exhibit high (V_{in} / V_{out}) ratios, although the correlation between the two factors is pending for futute investigation.

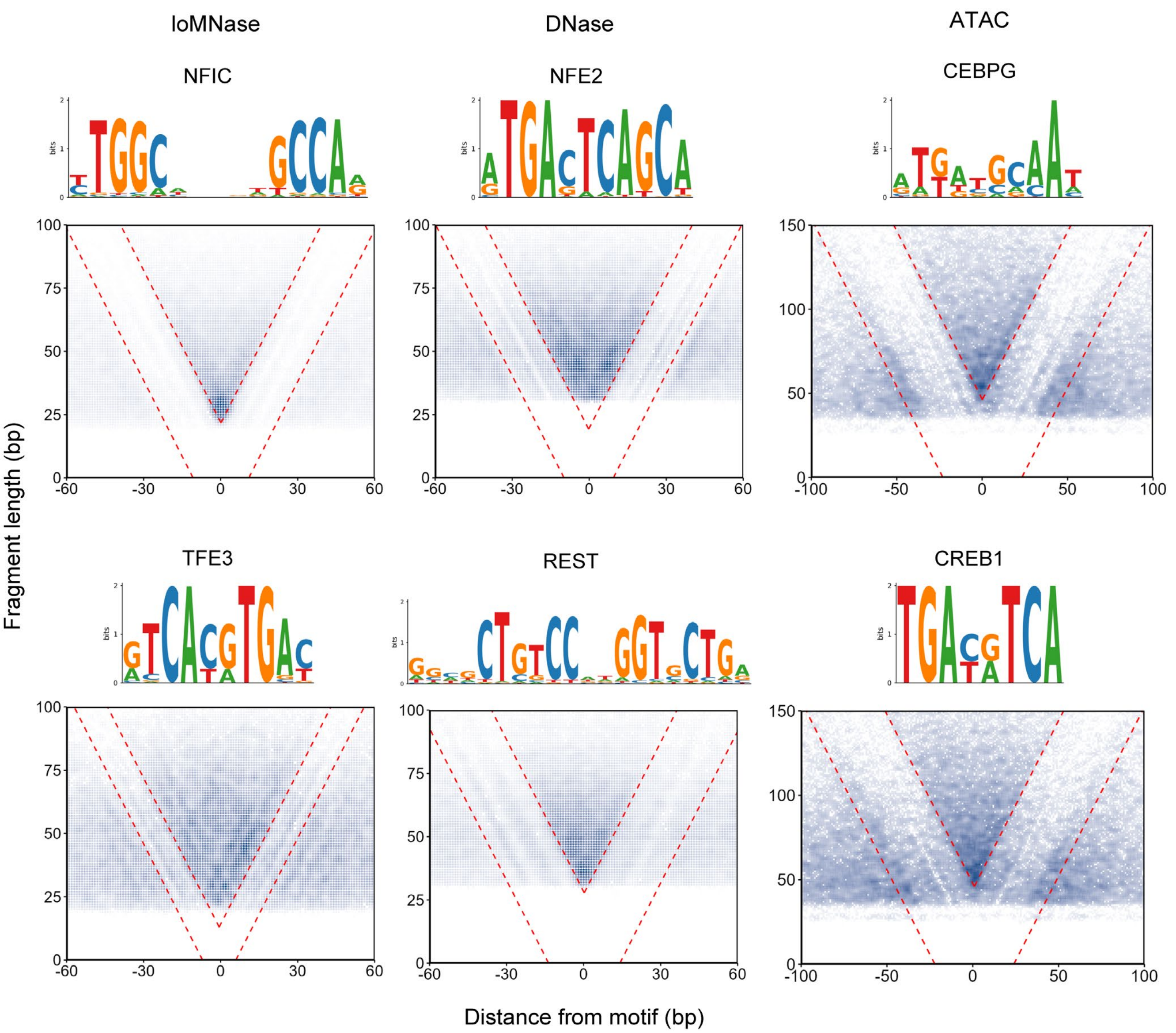

Minor:

Generally, more details on the methods would be beneficial: How were highly and weakly occupied CTCF sites defined in Figure 1? How was the in silico MNase digestion done? How were the MAZ occupancy levels defined?

We added descriptions on these topics to the Appendix file which includes the Methods section of this Correspondence article. These contents are also pasted below:

Definition of biased high or low occupancy CTCF motifs

Each CTCF motif is bidirectionally expanded to 100-bp window, annotated using CTCF N-ChIP signal, and ranked by average signal across the window. High or low occupancy CTCF motifs are defined as the top 25% or bottom 75% ranked motifs, respectively. The two groups of motifs are respectively intersected with flanking bias sequences from loMNase-seq, DNase-seq, or ATAC-seq data. The motifs with internal bias sequences are excluded from the two subsets.

in silico MNase digestion

A global fragment length distribution is established using all fragments in loMNase-seq dataset. The *in silico* digestion is conducted under the assumption that only fragments either including complete or without any remnant CTCF motif sequence are generated. Each fragment length is randomly sampled from the global fragment length distribution described above.

Calculation of MAZ (V_{in} / V_{out}) ratio and occupancy level

One inside-V and two outside-V areas are defined as shown in Fig2B. The inside-V area equals the sum of two outside-V areas. The number of dots (or fragments) within inside-V or outside-V areas is counted, and the ratio of (V_{in} / V_{out}) is calculated for each MAZ motif. MAZ motifs are categorized into five groups if the ratio falls within 1-3, 3-9, 9-27, 27-81, or 81-max ranges. MAZ occupancy level shown in Fig2B is expressed in RPM (reads per million) values using MAZ N-ChIP data.

Could there be an explanation added why motif sites with a juxtaposed bias sequence were analyzed and not all motif sites?

We added this phrase "To investigate if putative geometric differences can help distinguish V-shapes generated by TF occupancy or by bias sequences, we focused on sites with TF motifs closely juxtaposed with bias sequences" to the manuscript, before introducing three categories of sites for examination. Enforcing the occurrences of TF motif and bias sequence in close proximity controls for the same genomic background, and demonstrates that the V-shapes generated by TF occupancy can overwrite the V-shapes by bias sequences.

I couldn't find the datasets on GSA, could the accession codes be double checked?

Both accession numbers are released and publicly accessible. Our guess is that your might have searched the accession numbers in the GSA (Genome Sequence Archive) database, while we actually deposited the data to GSA-Human (Genome Sequence Archive for Human). The former is for deposition of data acquired from non-human species. In the revised manuscript, we added hyperlinks to the two accession numbers. The data can also be visited using links below:

HRA005744: https://ngdc.cncb.ac.cn/gsa-human/browse/HRA005744

HRA017804: https://ngdc.cncb.ac.cn/gsa-human/browse/HRA017804

I think the word "concerned" is not correctly used, e.g. 'further concerned about' should be 'expressed concern about' or 'were concerned about'.

Per your suggestion, we made the adjustment to the wording in the revised manuscript.

Code:

The GitHub hyperlink link is not working, but findable via the displayed text.

We checked and found it working in the submitted *.docx* file. The problem might have occurred in the pdf file conversion process.

While the GitHub folder structure makes it possible to navigate through the files, there is no README with an overall description.

We added README file to the repository.

Reviewer #2:

Summary

Zhang & Xu provide a compelling reply to Duran et al. (Genome Biol. 2026), who have recently provided seemingly straightforward but disputable evidence claiming that V-plots are not a reliable methodology to investigate protein-DNA interactions. Zhang & Xu clearly illustrate, both qualitatively and quantitatively, differences between "real" V-plot signals (obtained when focusing on TF binding motifs) and artifact V-plots (obtained when focusing on enzyme's bias motifs).

This manuscript supports the relevance of V-plots in epigenomic analysis workflows. This is of broader significance: conclusions from Duran et al. were raised questions about the validity of previously published results based on V-plot analyses. Zhang & Xu indisputably refute these conclusions to show that biologically relevant V-plots are valuable and can be quantified. Researchers who have used this approach in the past, or were intending to use it for future analyses, are thus reassured that V-plot-based interpretations remain methodologically sound and biologically meaningful, and can proceed with confidence in building upon or applying this framework in their epigenomic studies.

Thank you for your positive comments and the constructive suggestions on our work.

Major points

My main suggestion would relate to the size of the sequence the authors use to support their claim. They use 3bp-long sequence bias motifs for MNase, DNase or Tn5, and in fact recover the same type of non-significant V-plot signal (thin white lines, ratio of Vin/Vout of ~ 1). I would recommend generating V-plots centered on randomly chosen sequences with a length comparable to that of the TF motifs they are studying (7-16bp, according to Fig. 2D). Even though this would not directly address the question of the V-plot artifact emerging from sequence bias from enzymes, this would clarify the comparison between a well-resolved V-plot and a non-significant V-plot over motifs of the same size.

We appreciate your constructive suggestion on controlling the size of putative bias sequences. Using CTCF motif as an example, we generated 11 scramble "motifs" by shuffling the nucleotides without altering their weights at each position. Some motifs are shown below as examples:

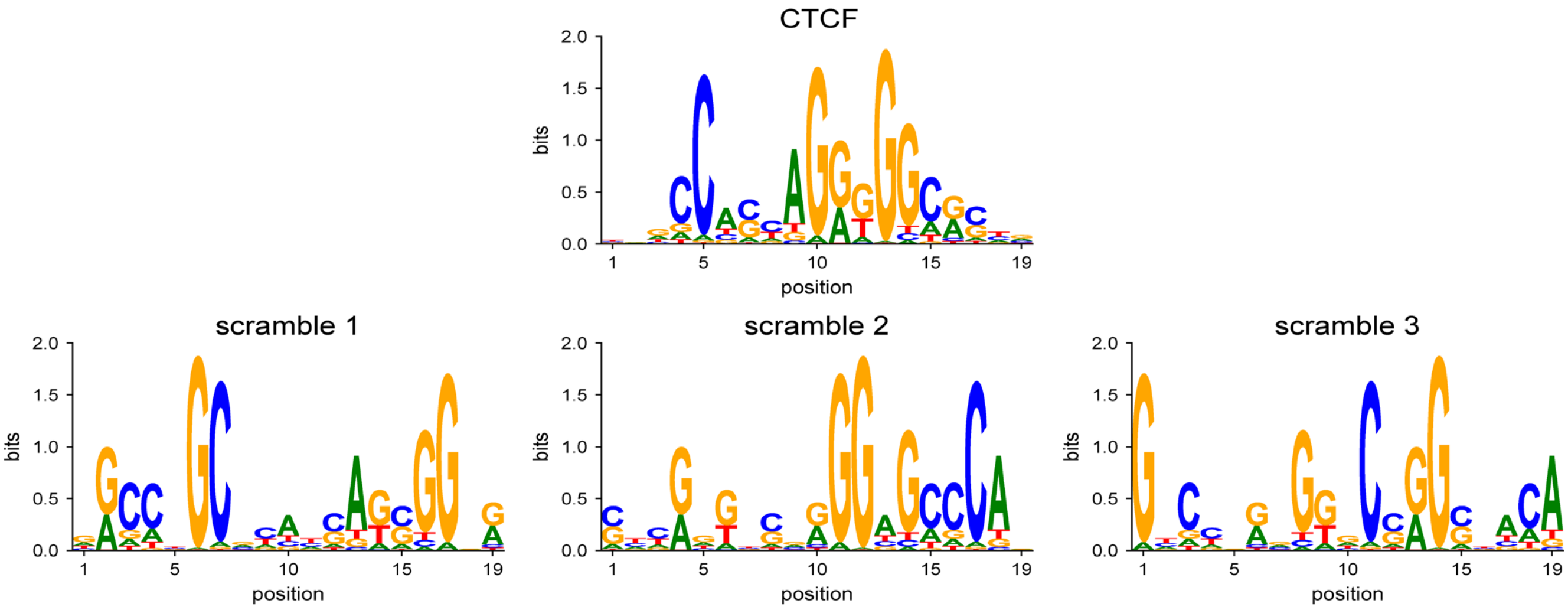

Using the pipeline we introduced above when addressing to Reviewer 1's comments, we show below (also as new FigS8) that although the V-shapes at scramble motifs may have V-channel width as long as 20 bp, the two types of V-shapes can indisputably be segregated using the (V_{in} / V_{out}) ratio as the parameter.

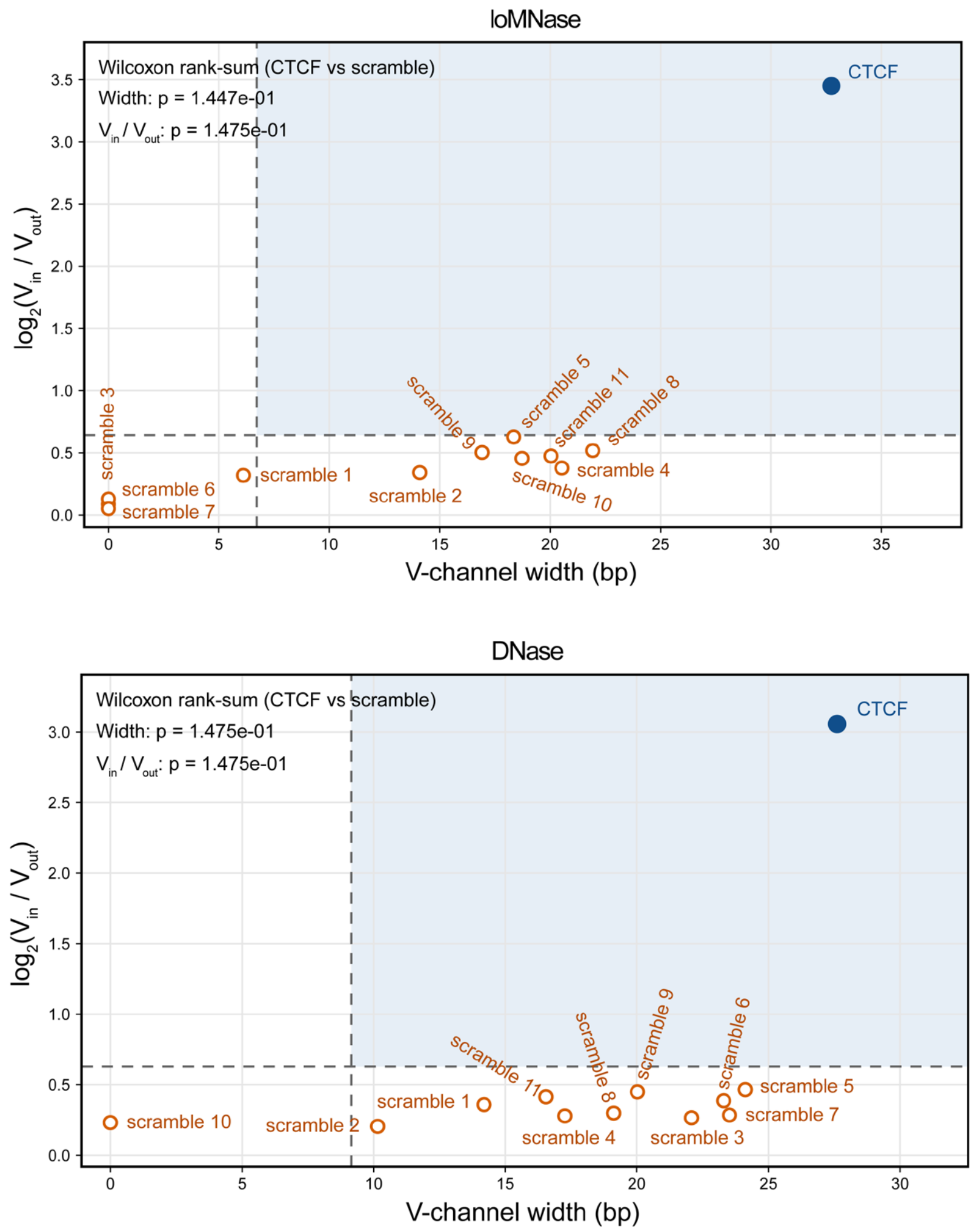

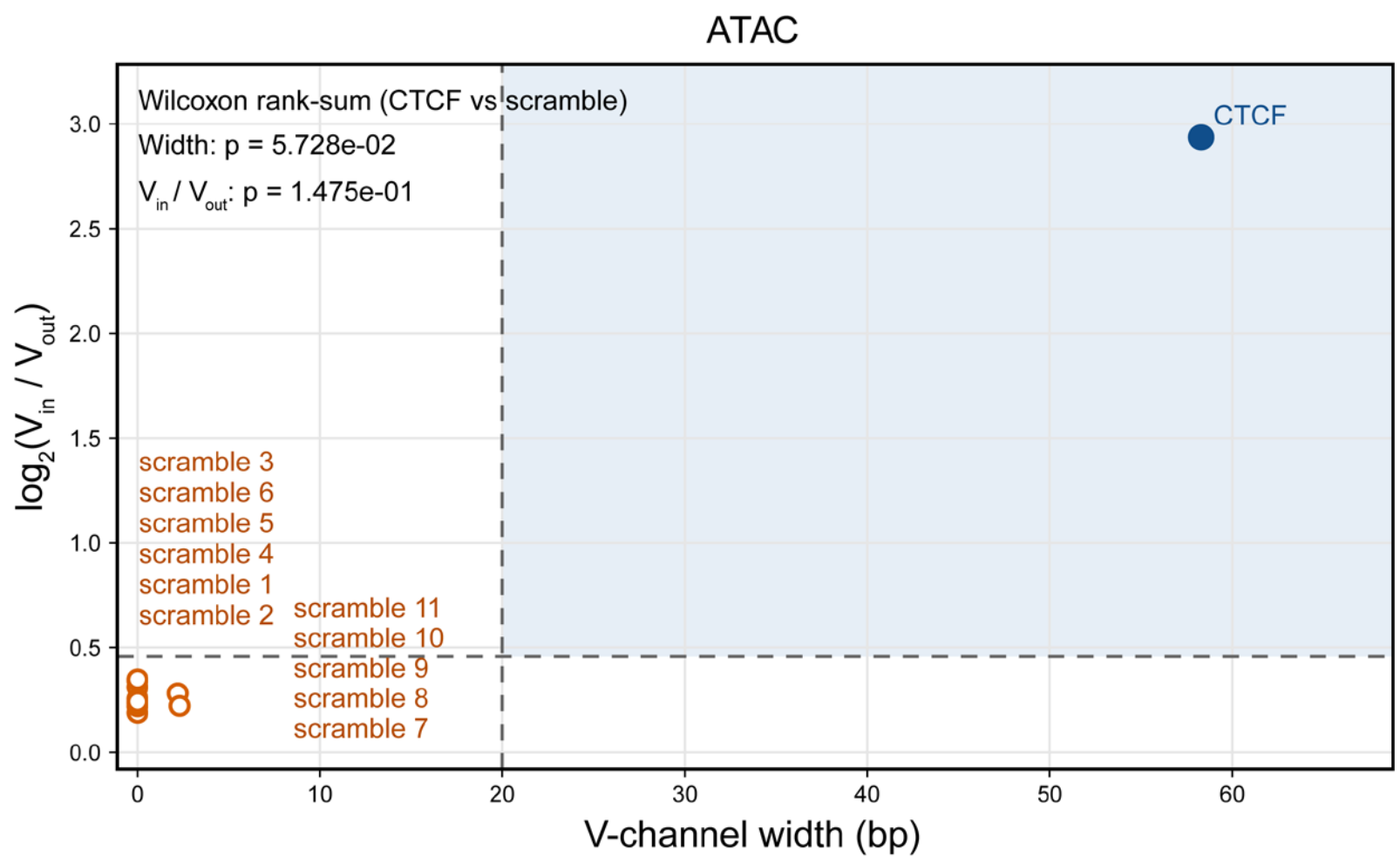

Although this goes beyond the review of this individual manuscript, I suspect that the MNase dataset generated in S. pombe by Duran et al., shown in their Fig. 1C, is in fact of insufficient quality to reliably identify V-plots. Duran et al. have also used a non-standard computational approach to annotate TF binding sites in the S. pombe genome. I believe it could be worth investigating the quality of their data, should the authors want to more directly and openly dispute conclusions from Duran et al.

The motifs used in Durán et al Fig1C were not clearly specified to be consensus sequences or occupied motifs annotated using ChIP data. Therefore, using ChIP data for three subunits (Cdc10, Res1, Res2) of the MBF complex from an S. pombe TF binding atlas (PMID: 40015273), we annotated the 882 consensus sequences in S. pombe genome, and divided them into 31 occupied and 851 un-occupied motifs (shown below).

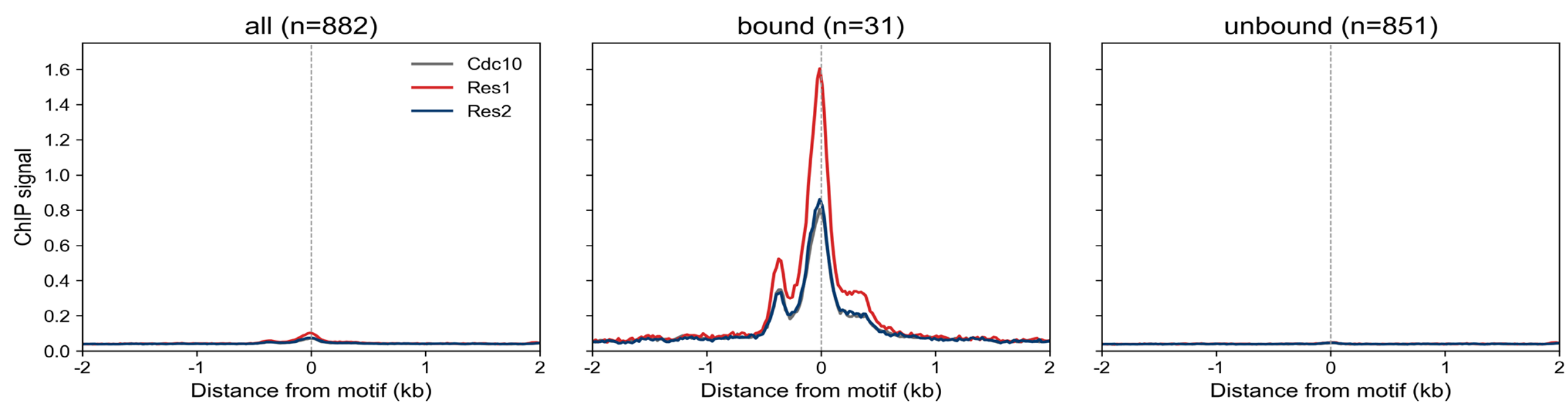

We next generated the V-plots as shown in Durán et al Fig1C using their MNase digestion data either on chromatin or on naked DNA. As shown below, only the set of all consensus motifs or the subset of un-occupied motifs can reproduce the V-shapes as in shown their Fig1C, suggesting that they may have used an un-annotated collection of motifs when doing V-plot analysis. Since the majority of the motifs are un-occupied (851 out of 882), MNase behaved in largely the same way at these sites on chromatin or on naked DNA in their digestion. So their analysis in Fig1C only demonstrates the existence of V-shapes, like what we show in our Fig1 for the bias sequences, but does not challenge the usefulness of V-plot in genuine TF footprint

discovery.

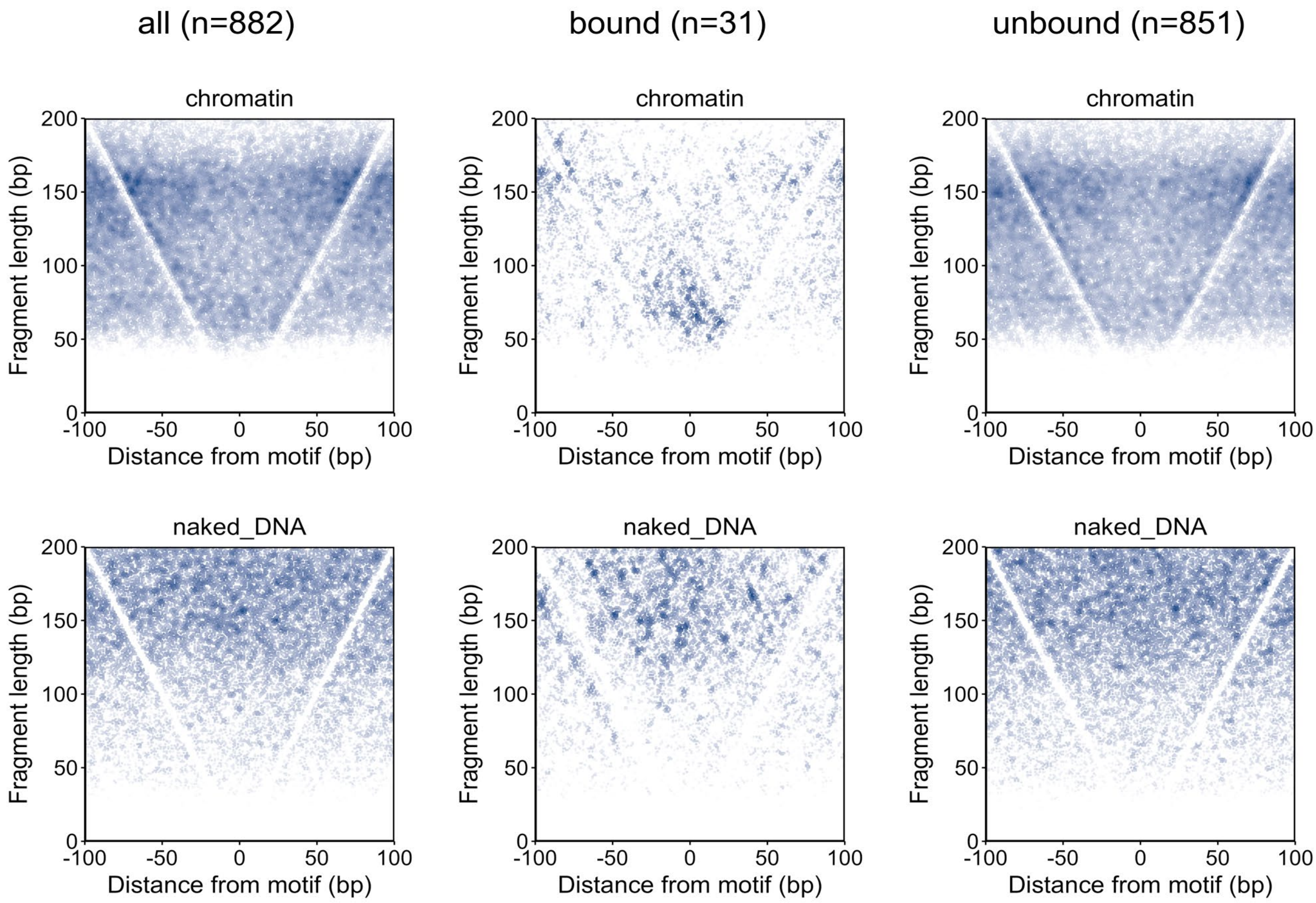

In the same vein, it would be important to specify which tools were used in this study for motif scanning (e.g., FIMO, MEME, others?), and exact scanning parameters, as these choices can significantly influence the resulting V-plot signals.

This manuscript was submitted as a "Correspondence" article which does not allow a Methods section in the main text. In the revised version, we include a Methods section and related references in the Appendix file.

Minor points

- A Methods section is missing. I understand that all analysis notebooks are publicly available, but I believe at least some basic information should be provided in a Methods section, at least to cite the libraries being used (e.g. bedtools, R, ggplot, etc) (and ideally their version).

This manuscript was submitted as a "Correspondence" article which does not allow a Methods section in the main text. In the revised version, we include a Methods section and related references in the Appendix file.

- Why is the schematic explanation of V-plots in Fig. 2A? I would suggest having it in main Fig.

1 or in first Fig. S1, to clearly introduce to the reader what the plots represent.

We accepted this suggestion and moved this diagram to FigS1 to help readers naive on V-plot understand this analysis in the beginning of the manuscript. But the sophisticated information in this diagram (V-channel width, footprint length, V_{in} / V_{out} enrichment, ...) is only mentioned in the second half of this manuscript when we introduce and benchmark the two graphical parameters.

Reviewer #3:

This commentary provides important insight and guidance for transcription factor footprint analysis using V-plots. The recent paper by Duran et al. suggested that a V-shaped pattern can be observed even in the absence of a true transcription factor footprint, due to sequence bias when motif-centered analysis is performed. The authors aim to provide criteria to distinguish artificial V-shaped patterns caused by sequence or enzyme bias from true transcription factor footprints. Using CTCF and other transcription factor motifs as examples, the authors demonstrate that a wider V-channel and inside-V enrichment are useful indicators for detecting true footprints. Although these criteria are potentially valuable, the results presented here remain largely qualitative. The manuscript does not provide statistical methods or quantitative measurements to support the conclusions or to standardize the proposed approach. Additional quantitative analyses would strengthen the claim that these features can reliably distinguish true transcription factor footprints from bias-driven artifacts. Other major concerns are summarized below.

Thank you for your time to review this manuscript and feedback us with helpful suggestions. In the revised manuscript, we provide an executable pipeline (deposited at https://github.com/diovs/V-plot-criteria/tree/main) to help putative users examine the V-shapes generated from their datasets using statistical methods. Please find details in our response to Reviewer 1, the Methods section, and the README file.

Line 79: The authors state: "In summary, we conclude that V-plot analysis using chromatin accessibility datasets, especially when in lack of robust ChIP data, should implement the V-channel width and inside-V enrichment as two essential criteria, to guarantee the validity of discovered TF footprints."

This strong claim requires additional supporting evidence and quantitative measurements with a statistical test. Although Figure 2D shows a clear separation between TF target sites and 3-mer sites, the analysis does not report variability across biological replicates or across different data sources. Because ATAC-seq is the most widely used method and appears to produce the least discrete V-plot pattern, ATAC-seq data should also be included in Figure 2D to assess

whether the observed separation is robust across assays.

In the revised manuscript, we provide an executable pipeline (deposited at https://github.com/diovs/V-plot-criteria/tree/main) to help putative users examine the V-shapes generated from their datasets using statistical methods. Please find details in our response to Reviewer 1, the Methods section, and the README file. Analysis using ATAC-seq data has been added as new Fig2E. In addition to compare V-shapes at TF motifs with the ones at 3-digit enzyme bias sequences, we also show that V-shapes at TF motifs can be statistically segregated from the ones at shuffled "motifs". Please find details in our response to Reviewer 2 and in FigS8.

Low-MNase-seq:

Low-MNase-seq is quite different from ATAC-seq and DNase-seq, and less popular compared to other methods. I am also not sure whether low MNase-seq should be categorized as a chromatin accessibility assay, although it has been used to capture MNase-sensitive nucleosomes and transcription factor footprints. The data from standard MNase-seq would also be informative.

We changed the wording from "chromatin accessibility" methods/datasets to "TF footprinting" methods/datasets, to more appropriately describe loMNase-seq's capability in resolving TF footprints than in measuring chromatin accessibility. As shown below, the peaks from the three datasets are highly overlapped with each other.

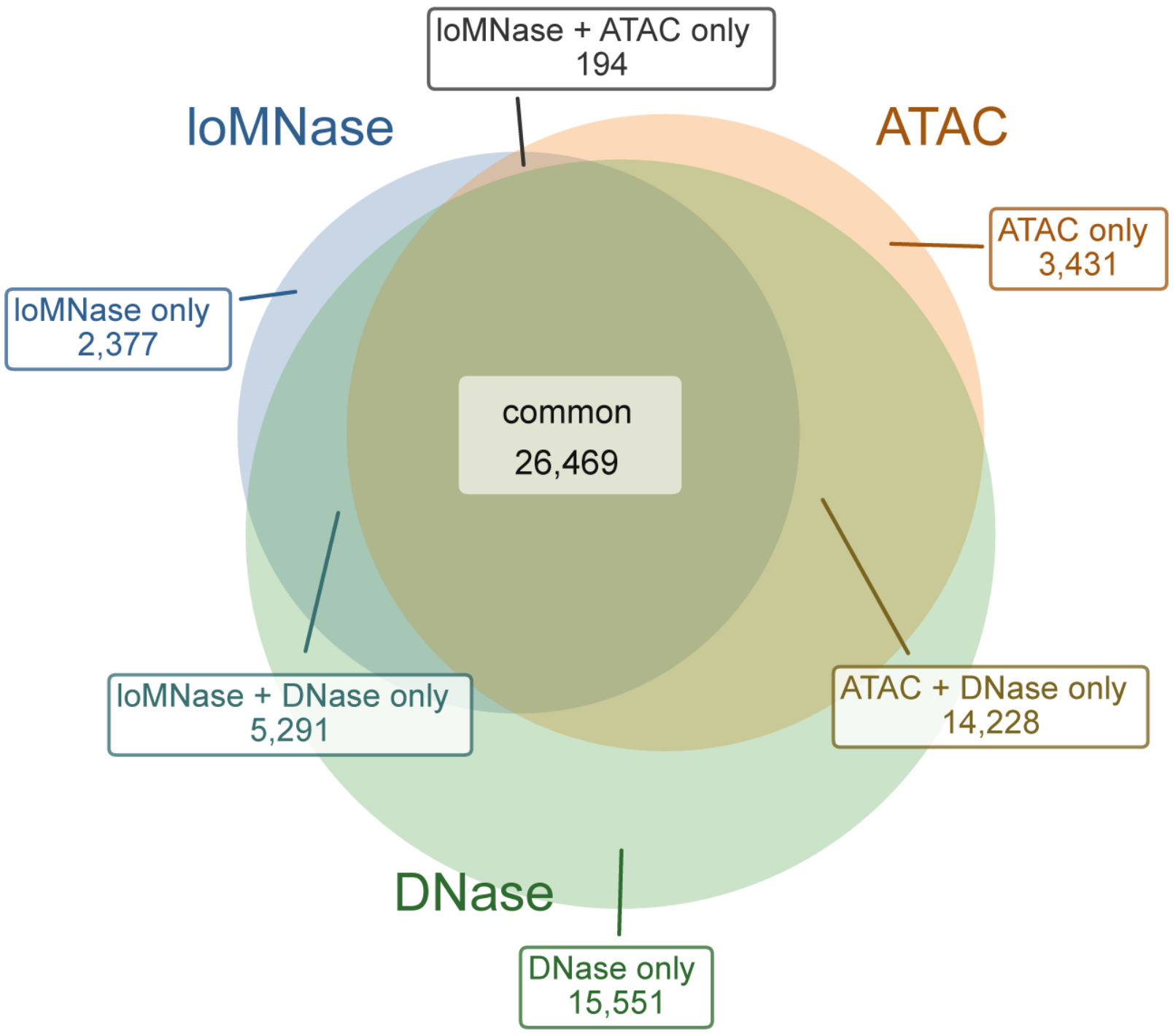

We also find that although the three datasets exhibit somehow different V-channel width for the same TFs (Fig2C-E), the (V_{in} / V_{out}) ratio is relatively constant between the three datasets (shown below). Both analyses suggest that loMNase-seq serves as a good option for TF

footprinting and V-plot analysis.

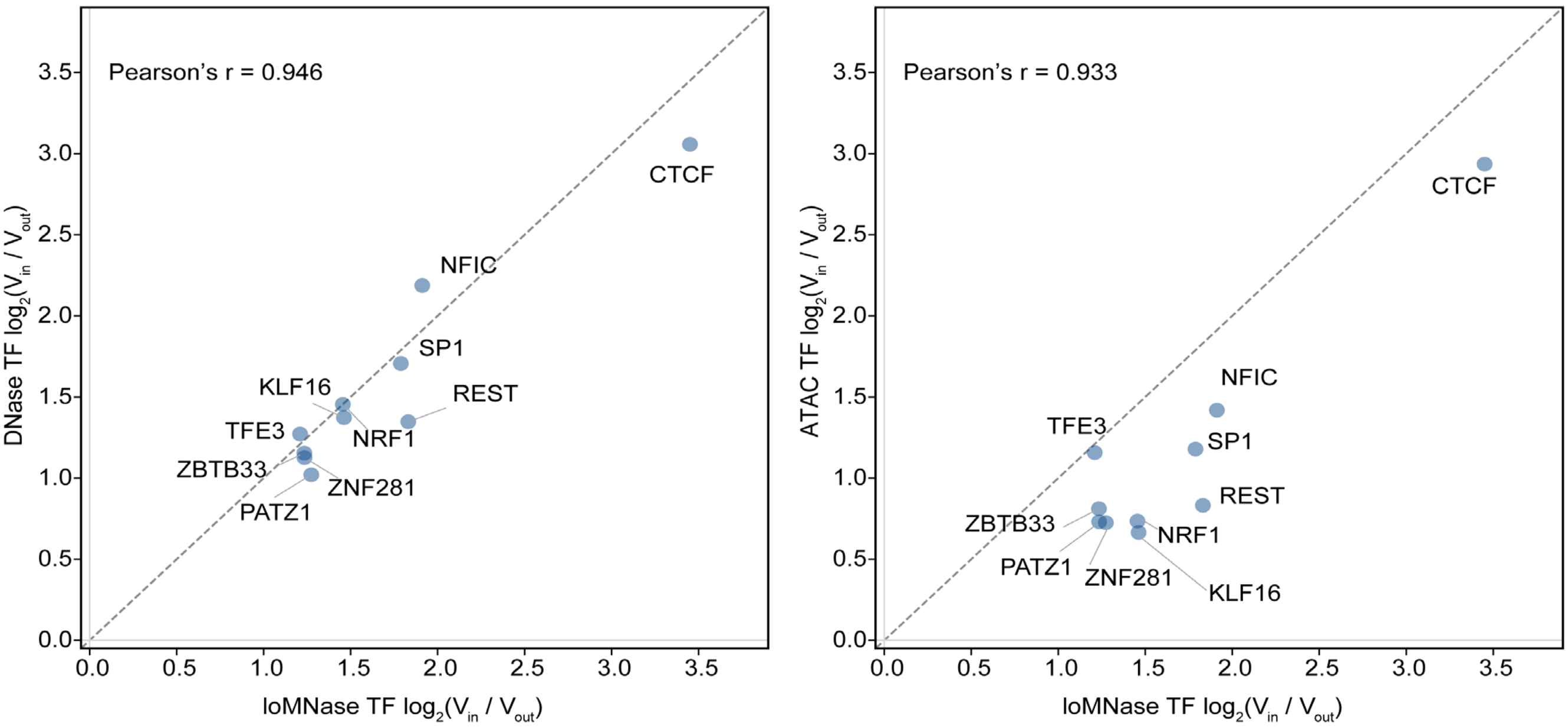

Using CTCF and MAZ as two examples, below we show side-by-side V-plots when using loMNase-seq or MNase-seq data. Although the regular MNase-seq data seems to uncover a similar V-channel and a comparable width, the other parameter, the (V_{in} / V_{out}) ratio, is drastically different between the two datasets. Regular MNase-seq data does not exhibit an obvious inside-V enrichment of fragments over outside-V fragments, probably due to harsh MNase digestion condition and the consequent loss of TF footprint fragments.

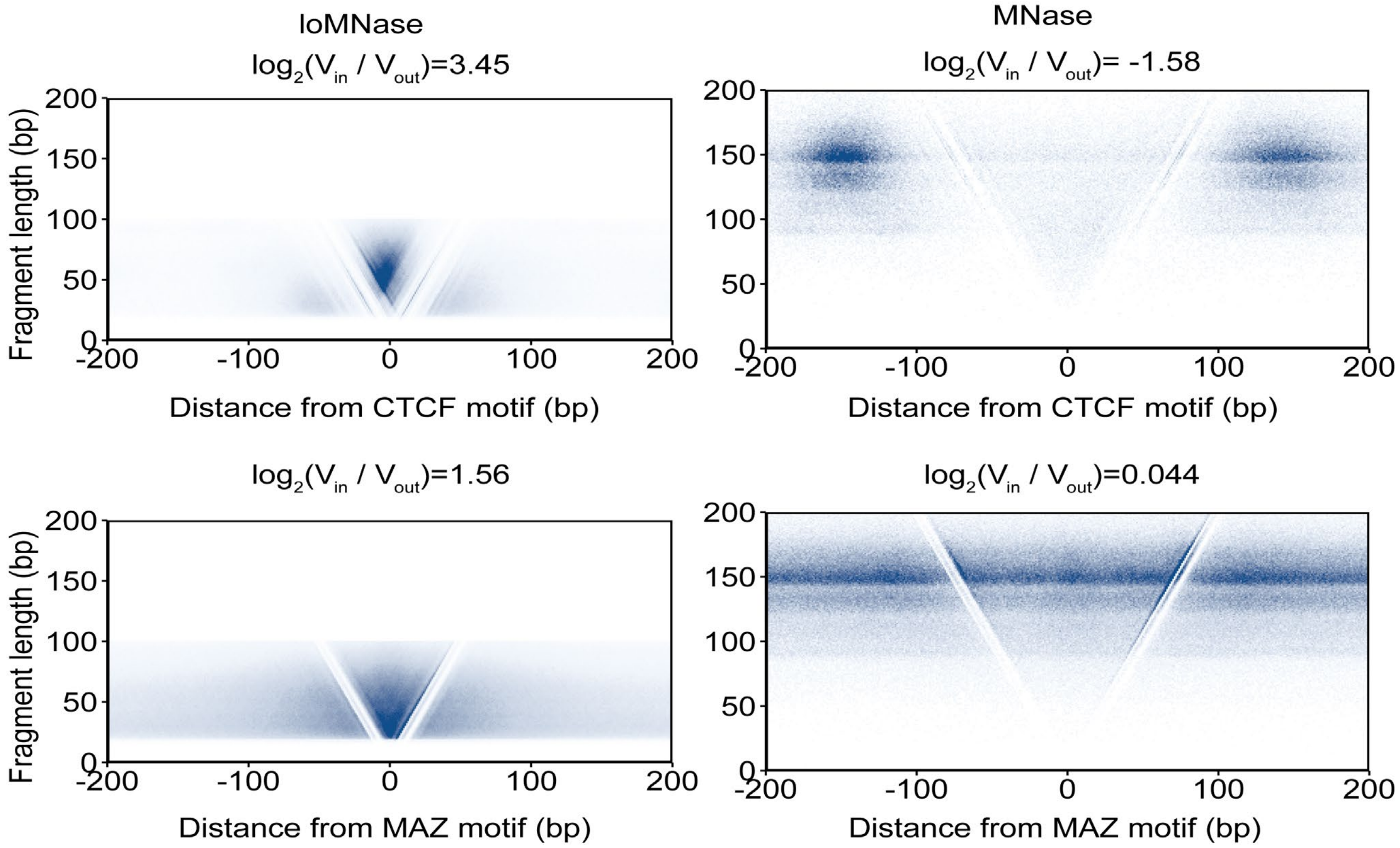

As shown in Supplementary Figure S2, the ATAC-seq data show weaker and more ambiguous

footprints or V-shaped patterns. By comparing these three panels, it still appears challenging to draw a definitive conclusion based solely on the suggested criteria of V-channel width and inside-V enrichment.

In applications of loMNase-seq or DNase-seq, MNase or DNase functions as exonuclease to chew DNA progressively, until the encounter with a resident TF. While in ATAC-seq, the Tn5-mediated transposition reaction does not have such a "progressive effect", possibly explaining why the V-shapes are relatively obscure in ATAC-seq data. Nevertheless, using statistical methods, our pipeline introduced above can succesfully segregate V-shapes at TF motifs from the ones at 3-digit enzyme bias sequences (Fig2E), or from the shuffled "motif" sequences (FigS8) when applied to ATAC-seq data. Therefore, the two graphical parameters we introduced in this manuscript stand as important criteria for TF footprint discovery in all three types of data.

Therefore, I believe the concerns raised by Duran et al. still provide an important caution for the field. V-shaped and enrichment patterns may differ depending on the combination of genomic data type, enzyme bias, and motif sequence composition. Sequence bias induced by MNase is also known to vary depending on MNase concentration.

We agree with you that Durán et al raised an important caution. Therefore, this manuscript is not making an argument with the results in Durán et al. Instead, we appreciate their timely bringing up the topic on V-shapes at bias sequences. Regarding diversity in bias sequences, in the revised manuscript, we demonstrate that using our pipeline, V-shapes at TF motifs can be statistically segregated from the ones at 3-digit enzyme bias sequences from all three assay types (Fig2C-E), or from the shuffled "motif" sequences (FigS8). Therefore, it is reasonable to think that the usage of the two graphical parameters we introduced here can be expanded to cases where subtle differences in bias sequences may occur under different enzyme digestion conditions.

Confirmation with other TFs and loci

It would also be useful for the authors to further clarify the choice of bias sites without motifs. The number of these negative control sites is also much larger than the number of CTCF binding sites, which may affect the comparison. For further validation, it would be useful to examine V-plots at genomic loci that contain CTCF motifs but are not bound by CTCF. Because MNase is known to prefer AT-rich sequences, it would also be important to assess V-plot bias at AT-rich sequences and at motifs for AT-rich sequence-binding transcription factors, as most of the transcription factors tested here appear to target GC-rich sequences.

1) In Fig1, FigS2, and FigS3, we used 100,000 randomly picked bias sequence sites because fragments from neither dataset are enriched at these sites. As shown below, using a

comparable amount of 1,000 bias sites makes it difficult to determine the existence of V-shapes at these sites.

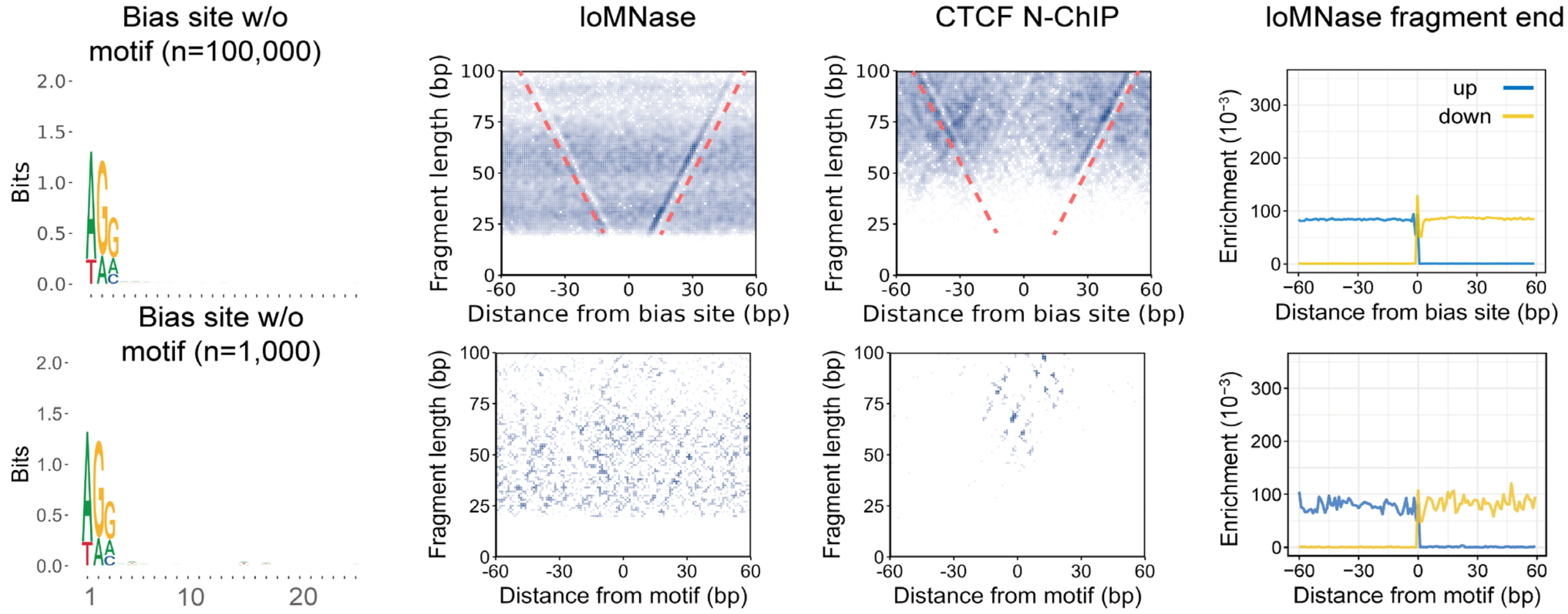

2) We generated a list of 25,615 un-occupied CTCF motifs (shown below, top left), and performed the same analysis using the pipeline we described above. The un-occupied CTCF motifs behave somehow like the scrambled "motifs", exhibiting shorter V-channel width and no enrichment over background (shown below, top right). The V-plots for occupied and un-occupied CTCF motifs are shown below (bottom row) to exhibit the differences in two parameters.

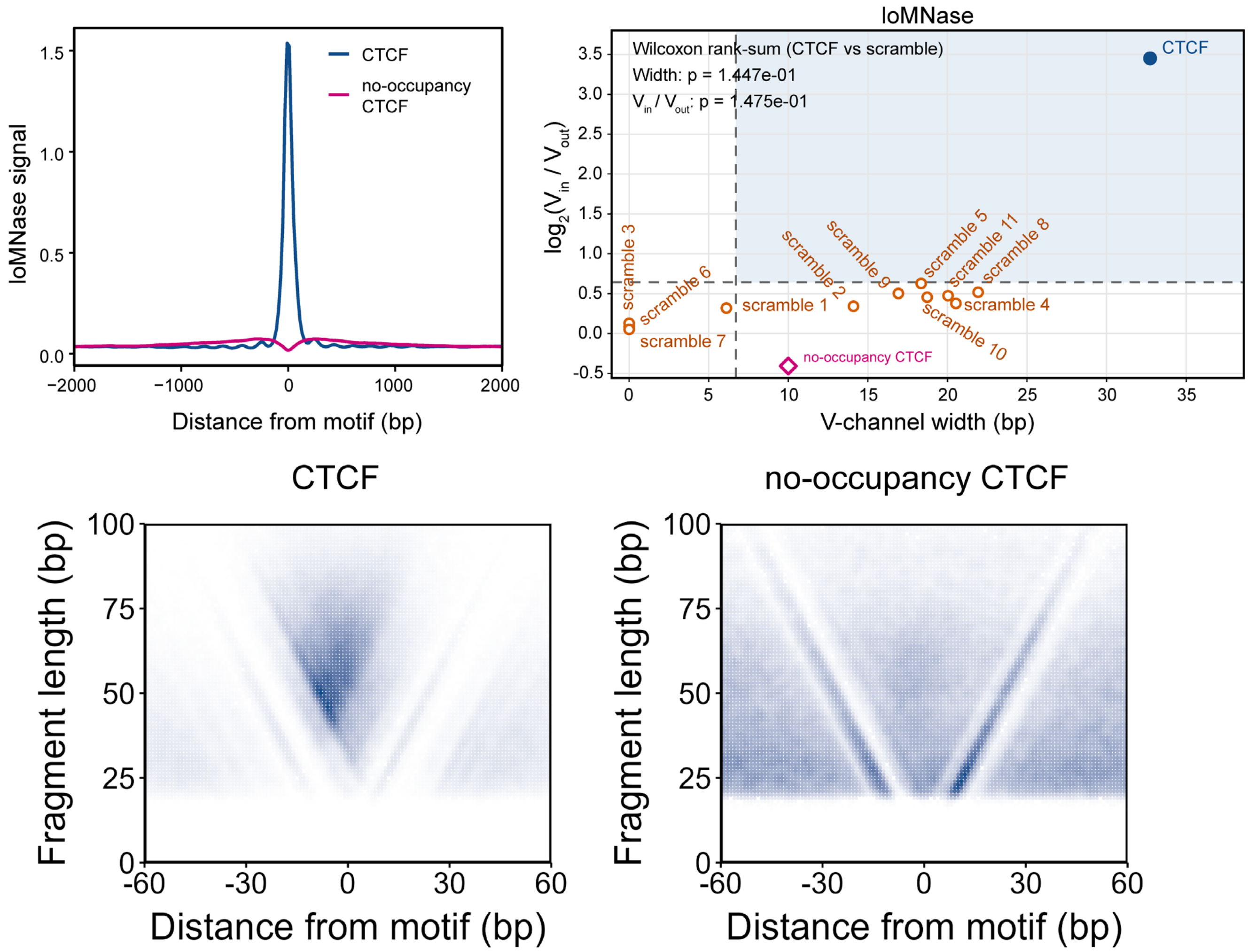

3) Regarding AT-rich sequences, in address to Reviewer 1 Major Comment 2, we provided some examples to show that TFs with AT-rich motif sequences exhibit wide V-channel and enriched inside-V over outside-V fragments as well.

5th Aug 2026

Manuscript Number: MSB-2026-13790R
Title: Two essential criteria for transcription factor footprint discovery using V-plot
Author: Qifan Zhang
Chenhuan Xu

Dear Dr Xu,

Thank you for the submission of your revised manuscript to Molecular Systems Biology. We have now received the enclosed reports from the referees that were asked to re-assess it. As you will see the reviewers are now globally supportive and I am pleased to inform you that we will be able to accept your manuscript pending the following final amendments and appropriate response to reviewers:

1) Please remove all figures from main manuscript file and leave only the main figure legends placed after the references.
2) Please ensure that the section order of the manuscript is corrected to the following: Title page - Main text - Acknowledgements (if any) - Disclosure and Competing Interests Statement - References - Figure Legend - Table (if any)
3) As part of the EMBO Publications transparent editorial process initiative (see our policy here: https://www.embopress.org/transparent-process#Review_Process), Molecular Systems Biology will publish online a Peer Review File (PRF) to accompany accepted manuscripts. This file will be published in conjunction with your paper and will include the anonymous referee reports, your point-by-point response and all pertinent correspondence relating to the manuscript. Let us know whether you agree with the publication of the PRF and as here, if you want to remove or not any figures from it prior to publication. Please note that the Authors checklist will be published at the end of the PRF.
4) Please note that we now mandate that all corresponding authors list an ORCID digital identifier. This takes <90 seconds to complete. We encourage all authors to supply an ORCID identifier, which will be linked to their name for unambiguous name identification.
5) Please provide a point-by-point letter INCLUDING my comments as well as the reviewer's reports and your detailed responses (as Word file).

I look forward to reading a new revised version of your manuscript as soon as possible.

Yours sincerely,

Poonam Bheda, PhD
Senior Scientific Editor
Molecular Systems Biology

Reviewer #1:

The authors fully addressed my comments. The methods are described in great detail, there is a statistical test on the V-lots, and the additional experiments further support their findings. The code is nicely documented.
I have two last points which can be seen more as suggestions:
First, the V-plots on the unoccupied CTCF motifs done in response to Reviewer 3 could be added as separate supplementary figure or added to supplementary figure 8. It's quite illustrative for the method.
Second, the code repository could benefit from example files to do a test run with. This could help getting the input format right and testing whether the code runs through and produces the expected results.

Reviewer #2:

The authors have addressed all my concerns and provided comprehensive answers to them. I have no further comment.

Reviewer #3:

All of my previous concerns have been addressed.

1) Although author contributions can be included for a Correspondence, the free text should be removed from the manuscript and specified as author contributions in our submission system using CRediT. CRediT has replaced the traditional author contributions section because it offers a systematic machine-readable author contributions format that allows for more effective research assessment. You are encouraged to use the free text boxes beneath each contributing author's name to add specific details on the author's contribution. More information is available in our guide to authors: https://link.springer.com/journal/44320/submission-guidelines#cms-Manuscript-organisation-and-formatting

We have removed this section from the revised manuscript.

2) A Data availability section can also be included in the Correspondence. Please note that the Data Availability section should only include data and code that have been generated in the course of the current study, not for previously published studies. Therefore please remove the statement on the loMNase-seq and N-ChIP which were published in your previous paper. The information on these datasets is appropriately referenced in the Methods. For the code in Github, please format the Data Availability statement according to the example below:

"The computer code produced in this study is available in the following database:

- Modeling computer scripts: GitHub

(https://github.com/SysBioChalmers/GECKO/releases/tag/v1.0)

- [data type]: [full name of the resource] [accession number/identifier] ([doi or URL or

identifiers.org/DATABASE:ACCESSION])"

We deleted the statement on HRA005744, but kept HRA017804 because it was generated during this study. This section has been formatted per your suggestion.

13th Aug 2026
Manuscript number: MSB-2026-13790RR
Title: Two essential criteria for transcription factor footprint discovery using V-plot

Dear Dr Xu,

Thank you again for sending us your revised manuscript. We are now satisfied with the modifications made and I am pleased to inform you that your paper has been accepted for publication.

Your manuscript will be processed for publication by EMBO Press. It will be copy edited and you will receive page proofs prior to publication. Please note that you will be contacted by Springer Nature Author Services to complete licensing information.

Should you be planning a Press Release on your article, please notify the production team via this form in order to coordinate publication and release dates: https://forms.cloud.microsoft/e/eH4A8JVP6g

If you have any questions, please do not hesitate to contact the Editorial Office. Thank you for your contribution to Molecular Systems Biology.

Yours sincerely,

Sincerely,

Poonam Bheda, PhD
Scientific Editor
Molecular Systems Biology

>>> Please note that it is Molecular Systems Biology policy for the transcript of the editorial process (containing referee reports and your response letter) to be published as an online supplement to each paper. If you do NOT want this, you will need to inform the Editorial Office via email immediately. More information is available here: https://link.springer.com/partners/embo-press/editorial-policies#Peer%20review
